# Comparative evaluation of screw loosening and joint integrity across different implant-abutment connection types utilizing stock and CAD/CAM abutments under cyclic loading

**DOI:** 10.1186/s12903-026-08223-8

**Published:** 2026-04-13

**Authors:** Mohamed E. Gomaa, Atya A. El Gendy, Fatma A. El Hadad

**Affiliations:** Prosthodontic Department, Faculty of Dentistry, Tanta University, Tanta, Egypt

**Keywords:** Implant, Abutment, Internal connection, Torque loss, Screw loosening, CAD/CAM

## Abstract

**Background:**

Screw loosening is a common complication in dental implants, compromising their long-term stability and success. Implants with internal connection play a critical role in maintaining the structural integrity of implants by reducing the possibility of screw loosening.

**Purpose:**

To study torque loss in two different types of internal implant-abutment connections utilizing stock & custom-made abutments under application of cyclic loading.

**Materials and methods:**

Two groups were used; each group had sixteen epoxy-resin blocks divided into eight block in each subgroup. Implants with an internal implant abutment connection (hexagonal & trilobe) were installed in the epoxy-resin blocks, after abutment installation scanning was done and the crown was fabricated via CAD/CAM technology. After cementation of the crowns, the initial removal torque value was recorded via a digital torque gauge, and each block was prepared with an acrylic base to fit in the chewing simulator to apply dynamic cyclic loading. After the application of cyclic loading, the post load removal torque value was recorded. The removal torque loss ratio of the abutment screw before and after dynamic cyclic loading was subsequently calculated to evaluate the screw joint stability via removal torque measurements.

**Result:**

There was a significant difference in the amount of torque loss between the two types of internal connections.

**Conclusion:**

There was removal torque loss in both types of internal implant-abutment connections, demonstrating that the internal hexagon connection exhibited significantly lower torque loss compared to the internal trilobe connection in the tested groups.

**Clinical implication:**

Stock abutments offer superior mechanical stability and a significantly lower incidence of screw loosening under occlusal loading compared to custom-made alternatives. Furthermore, the hexagonal internal connection demonstrates enhanced structural integrity over the trilobe configuration, making it the preferred choice for long-term prosthetic success and reduced maintenance in high-load dental restorations.

## Introduction

Dental implants are widely utilized in the oral rehabilitation of single, partial, and total edentulism, facilitating the restoration of essential functions including mastication, deglutition, and phonetics while optimizing esthetics and patient comfort [1].

Unlike traditional restorative modalities, implant-supported prostheses offer superior stability and retention while preserving the integrity of adjacent dentition by eliminating the need for abutment reduction inherent in fixed partial dentures [2, 3].

Despite high success rates, implant failure may arise from biological factors such as poor oral hygiene, suboptimal bone density, and systemic comorbidities, or from biomechanical complications [4]. Among the latter, screw loosening and fracture represent prevalent clinical challenges. These failures are often precipitated by inadequate preload (tightening torque), settling effects, vibrating micromovements, and fatigue resulting from excessive bending moments [5] .

The implant-abutment connection (IAC) serves as the critical interface of the implant system. The geometries of these connections have evolved to enhance mechanical, biological, and esthetic outcomes, categorized primarily into external and internal configurations based on the geometric index’s relationship to the implant’s coronal platform [6, 7], Optimizing stress distribution at the IAC is fundamental to mitigating mechanical failures, including abutment or screw fractures, loosening, and microleakage [8] .

Internal implant-abutment connections generally exhibit superior esthetic profiles, increased joint strength, enhanced microbial seals, and improved long-term stability of the implant-abutment complex compared to external connections [9].

Internal interfaces are further classified into slip-fit (non-conical) or friction-fit (conical) geometries [10]. Common slip-fit designs include the internal hexagon and the trilobe connection. The trilobe architecture utilizes a three-point internal tripod design, allowing for abutment indexing at 120 degree intervals [11, 12]. This configuration provides high-precision anti-rotational stability and a robust press-fit, ensuring durable prosthetic replacement for single crowns and bridges [13].

Conversely, the six-point internal hexagonal connection offers enhanced tactile feedback during abutment seating and superior anti-rotational resistance [14], By protecting the fastening screw from lateral flexion forces, this geometry significantly limits screw loosening [15]. The hexagonal design permits abutment indexing at 60 degree increments, providing six distinct rotational positions [12, 16, 17].

The integration of Computer-Aided Design and Computer-Aided Manufacturing (CAD/CAM) has revolutionized implant prosthetics [18]. The prosthetic abutment has evolved from modified prefabricated “stock” abutments to fully individualized components customized via CAD/CAM protocols [19].

Contemporary workflows allow for the milling of customized titanium abutments from either prefabricated “pre-milled” blanks or directly from titanium discs. The latter involves the complex milling of the implant interface itself, a task requiring advanced CAM software and high-level technical expertise [20]. According to Al Fadda et al. [21]& Drago et al. [16], these digital workflows have effectively superseded traditional waxing, casting, and finishing techniques, thereby eliminating the dimensional inaccuracies and human errors associated with analog processes.

A screw joint comprises two components maintained in apposition by a threaded fastener [22]. The application of tightening torque induces screw elongation, generating tension. The subsequent elastic recovery of the screw pulls the implant and abutment together, establishing a clamping force [23], This force is distributed across the interface of the abutment, the screw threads, and the internal implant threads [24]. Joint stability is maintained as long as the clamping force exceeds the external joint-separating forces [25]. To prevent loosening, the magnitude of these separating forces must remain below the threshold of the established preload. Consequently, the two primary objectives in maintaining screw joint integrity are the maximization of clamping force and the minimization of separating forces [26].

To evaluate screw joint stability under simulated masticatory function, zirconia crowns are frequently utilized due to their high accuracy of fit, marginal adaptation, and optimized quality [27–29].

Dynamic cyclic loading via a chewing simulator is employed to replicate the complex vectors and magnitudes of occlusal forces, which are the primary catalysts for screw loosening [30–33], Eccentric loading, in particular, significantly influences the stability of the implant abutment connection [34, 35].

A digital torque gauge was employed to ensure the precise and reproducible application of torque to each abutment screw [36]. To facilitate retrievability and ease of access to the screw, a hybrid (screw-cement-retained) approach may be utilized. In this configuration, the crown is cemented to the abutment, while the abutment is secured to the fixture via the screw, allowing for simplified access through a designated screw channel [37].

The efficacy of the screw joint is quantified by calculating the ratio of removal torque (RMT) to initial tightening torque. Analyzing these ratios pre-loading vs. post-loading provides a critical indicator of the degree of loosening induced by dynamic mechanical stress [38].

The purpose of this study was to evaluate screw loosening and joint stability across different implant-abutment connection types utilizing stock and custom-made abutments under cyclic loading.

The null hypothesis is there is no statistically significant difference in torque loss between internal hexagonal and internal trilobe connections, regardless of whether a stock or custom-made abutment is utilized.

## Materials and methods

### Materials

The materials used in this study are categorized by their application in the implant and restorative workflow:

#### Implant and abutment components

- Implants: Replant/Legacy systems (Implant Direct, USA).
- Stock Abutments: Matched titanium stock abutments (Implant Direct, USA).
- Custom Abutment Material: Titanium blocks (DESS™, Alien Milling Technology, USA).
- Scan Bodies: Scan Adaptor (Implant Direct, USA).

#### Restorative and laboratory materials

- Base Material: Epoxy resin (CMB, Egypt).
- Guide Material: Clear acrylic resin (Acrostone, Egypt).
- Restorative Material: LAVA Plus Zirconia Blocks (3 M, USA) for crown fabrication.
- Luting Agent: Breeze resin cement (Pentron, USA).
- Access Closure: Estelite composite resin (Tokoyama, Japan) for sealing screw access holes.

#### Digital and hardware equipment

- Scanning: Swing laboratory scanner (DOF, Korea).
- CAD Software: Exocad Dental CAD (exocad GmbH, Germany).
- Cyclic Loading: CS-4.8 Chewing Simulator (SD Mechatronik, Germany).
- Measurement: HTG2-200Nc Digital Torque Gauge (IMADA, Japan).

### Grouping and sample size of the study

Two groups were used each group have sixteen epoxy-resin blocks divided into eight block in each subgroup.

Group (I): Implants with Internal Trilobe connection.

- Subgroup Ia: Implants with stock abutment.
- Subgroup Ib: Implants with custom-made abutment.

Group (II): Implants with Internal Hexagonal connection.

- Subgroup IIa: Implants with stock abutment.
- Subgroup IIb: Implants with custom-made abutment.

The samples were collected on the basis of a previous study. The significance level was 0.05,the power sample size was more than 80% for this study, the confidence interval was 95%, and the actual power was 96.11%.

The sample size was calculated via the computer program G power version 3.

The formula for sample size.

$$sample size (n)=\:\frac{({{\sigma\:}_{1}}^{2}\:+\:{{\sigma\:}_{2}}^{2})\:(\:{Z}_{\alpha\:/2}\:+\:{Z}_{\beta\:})2}{{(\varDelta\:\:-\:\varDelta\:0)}^{2}}$$

where:

n = Total sample size required per group.

$\:{{\sigma\:}_{1}}^{2},\:{{\sigma\:}_{2}}^{2}$ = Standard deviations of the two comparison groups.

$\:{Z}_{\alpha\:/2}$ = The critical value for the significance level (alpha 0.025), (Z(0.025) = 1.96).

$\:{Z}_{\beta\:}=$ The critical value for the desired power (typically 0.84 for 80% power).

∆= The expected mean difference between groups.

$\:\varDelta\:0=\:$The difference under the null hypothesis (usually 0).

Based on the power analysis using the formula above, a total sample size of n = 32 was determined (16 specimens per group) to ensure a power of 80% at a 5% significance level.”

### Methods

#### Implants installation

**Fig. 1:**
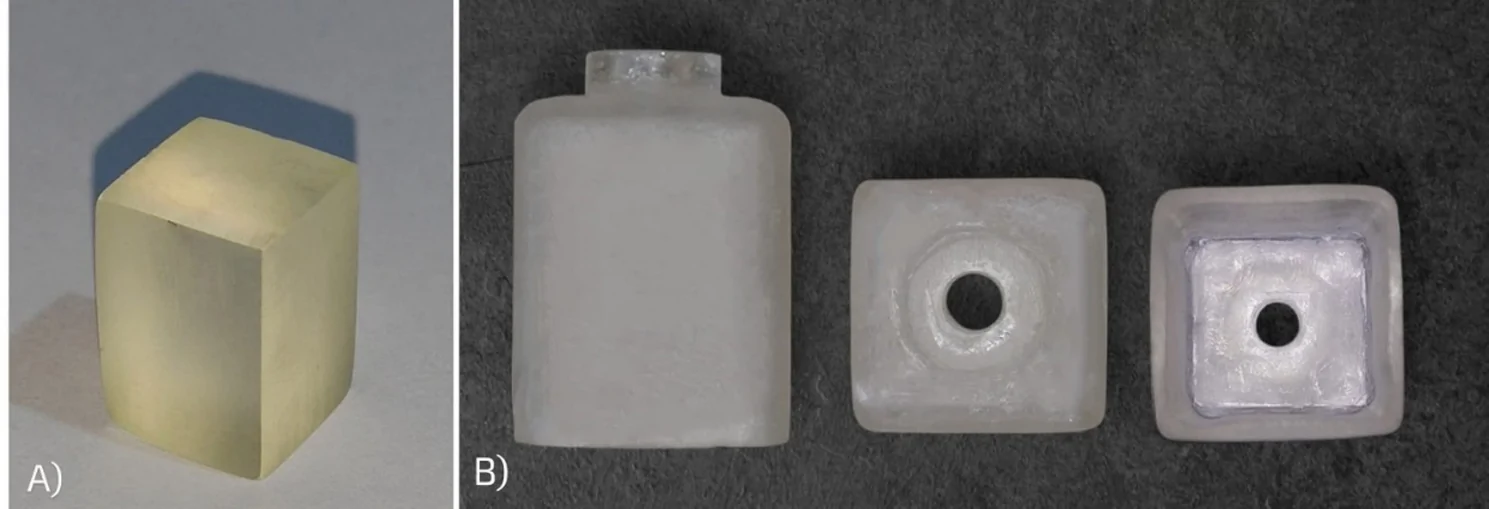
Showing. **A** Epoxy resin block. **B** Clear acrylic resin guide in which the epoxy block will be fit in

**Fig. 2:**
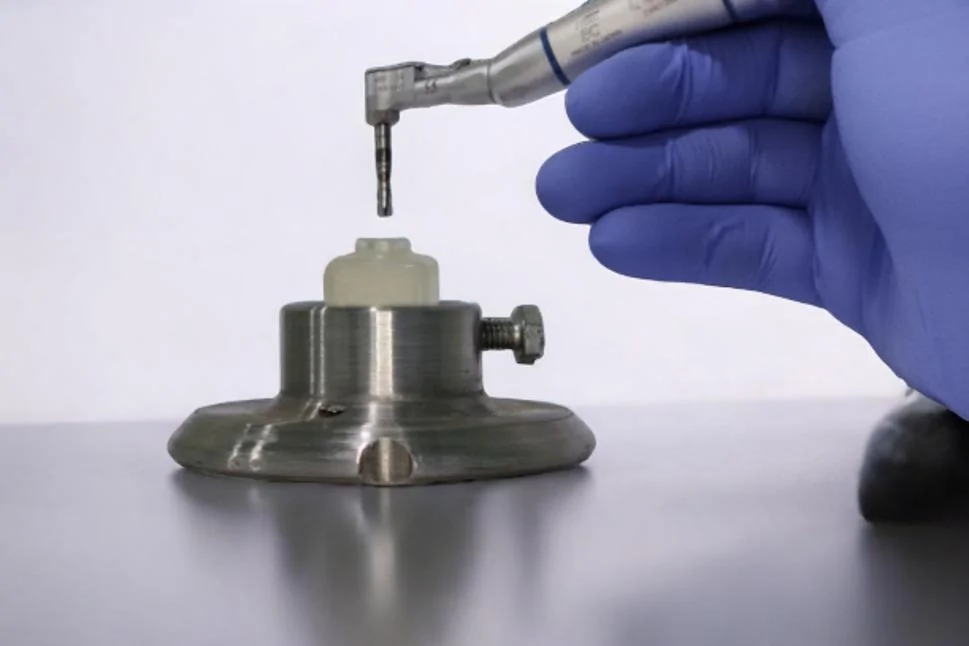
Drilling in the epoxy blocks using the resin guide fixed in a metal base

In each group sixteen identical cuboidal blocks were made from epoxy resin material and divided into two subgroups, each block received an implant via a resin guide fixed in a metal base [39] (Figs. 1 and 2). The implants were embedded in the epoxy resin blocks using a vertical positioning device to ensure axial alignment. Each implant was placed to a depth where the implant platform (shoulder) was flush with the superior surface of the resin block, simulating crestal bone level. This ensured that the entire length of the implant body was supported by the resin and that the height of the suprastructure (crown-abutment complex) remained consistent for all specimens, thereby standardizing the lateral forces applied during cyclic loading [40] (Table 1).

**Fig. 3:**
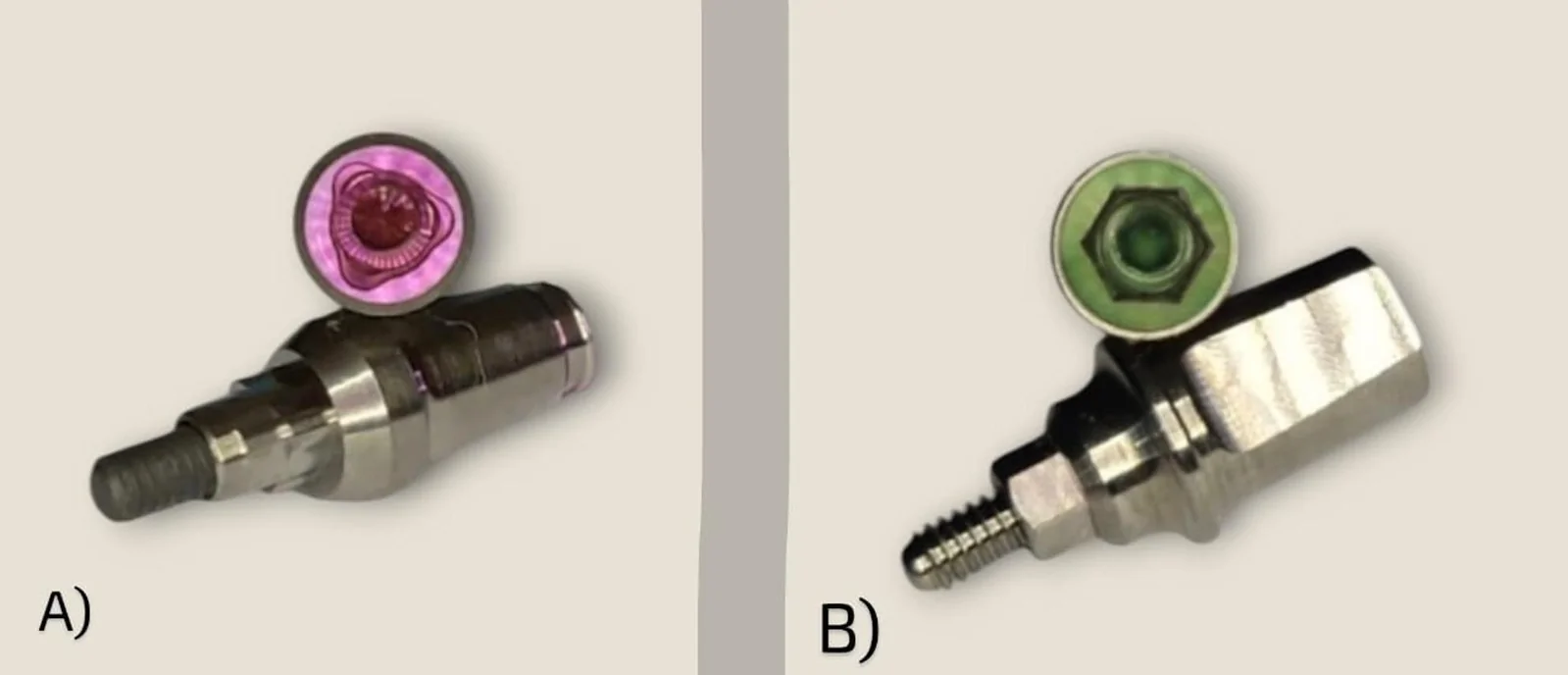
Implants showing the connection with their matched titanium abutments. **A** Implant with internal trilobe connection. **B** Implant with internal hexagon connection

For both groups, implants 13 mm in length and 4.2 in diameter were used (Fig. 3).

- Group (I): Implant with an internal trilobe implant-abutment connection.
- Group (II): Implant with an internal hex implant-abutment connection.

The abutments were selected with identical dimensions and geometries, with the sole difference being the interface design for connecting to the implant.

#### Designing of Custom CAD/CAM abutment

**Fig. 4:**
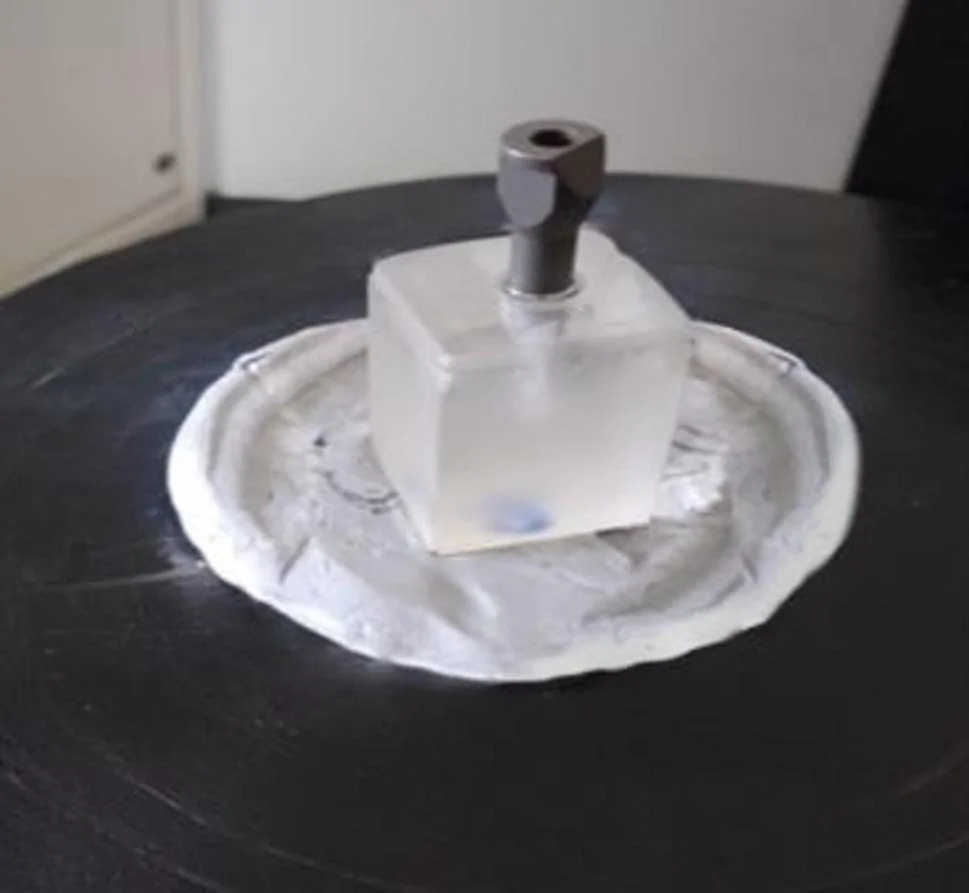
Scan body fixed to implant. Adapted from Mostafa et al. [40]

**Fig. 5:**
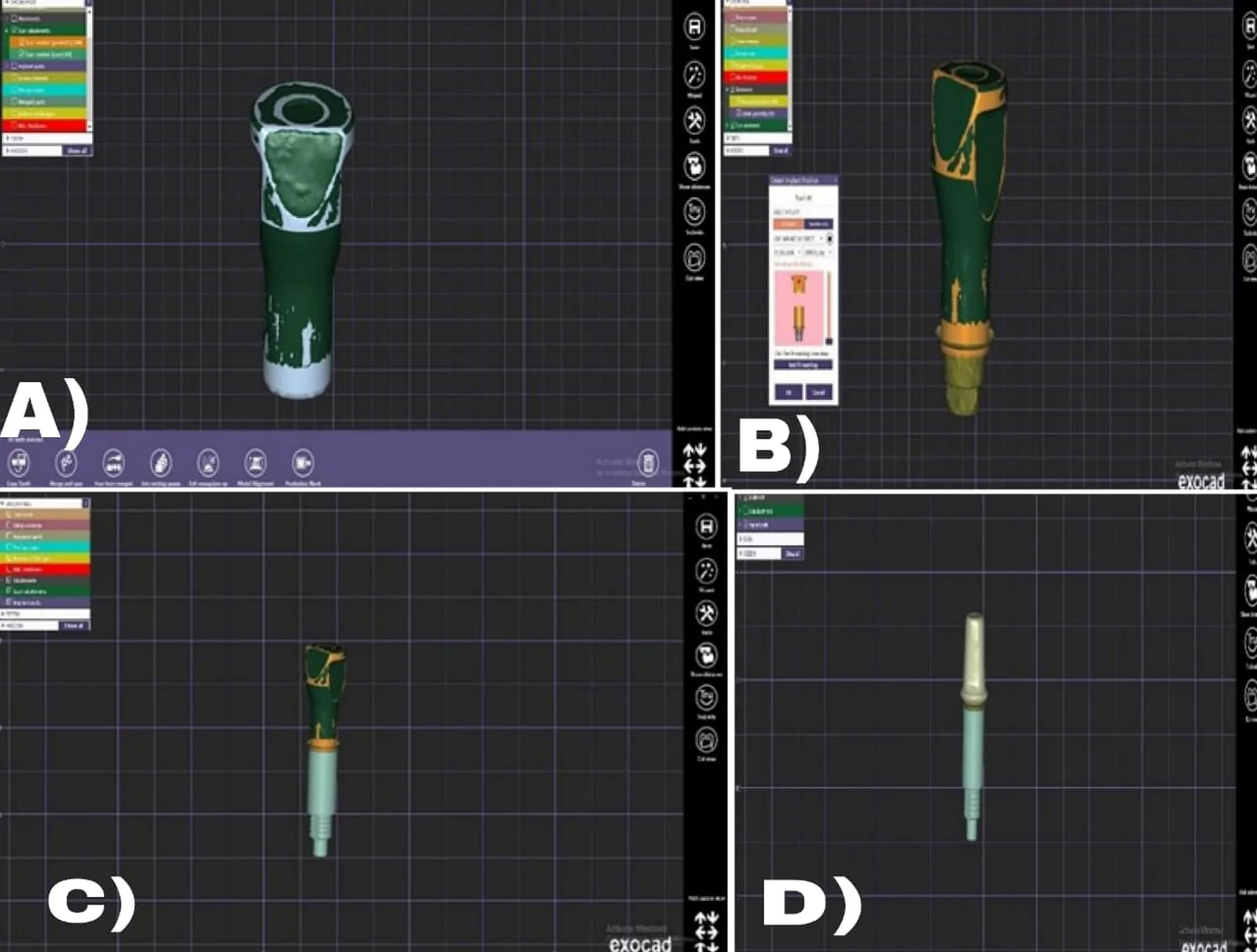
Digital workflow for custom-made abutment. **A** The STL file of the Scanbody. **B** Recognition of implant-abutment connection from the library of exocad. **C** Designing of CAD/CAM abutment. **D** The final design of the abutment

The digital workflow for abutment fabrication followed a protocol previously reported by Mostafa et al. [40]. After implant installation in the epoxy blocks, a scan body was screwed into the implant and scanned using a laboratory scanner to detect its shape and dimension and the connection of implant (Fig. 4), then the scan was exported to the CAD software to design the abutment by merging between the two files of scan from scan body and file of stock abutment scan. (Fig. 5).

Eight abutments were designed in same shape, length, same collar height and the same connection according to the dimensions of prefabricated stock abutment by cad software for standardization in this study.

**Fig. 6:**
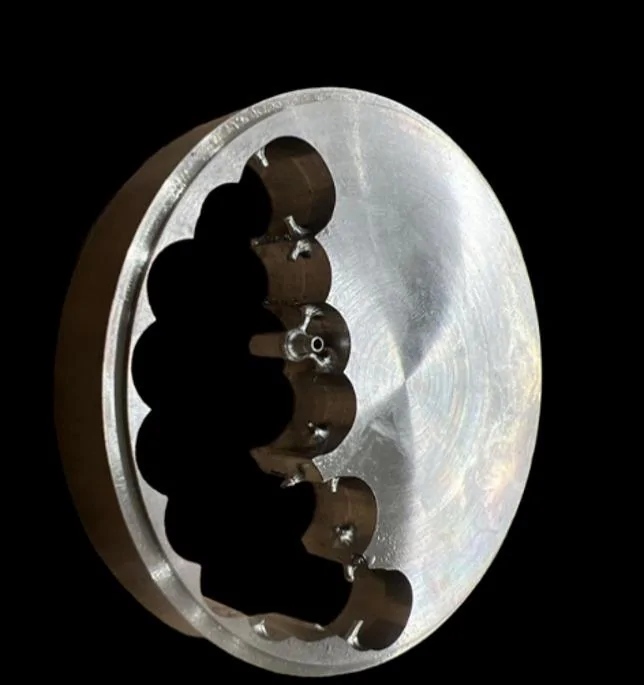
CAD / CAM milled abutment in titanium block. Adapted from Mostafa et al. [40]

The STL files of custom-made abutment were loaded into the CAM software that allows direct milling of the connection in a titanium disc (Fig. 6) by milling machine, then the customized abutments that obtained by this milling procedure were fixed to implant.

**Fig. 7:**
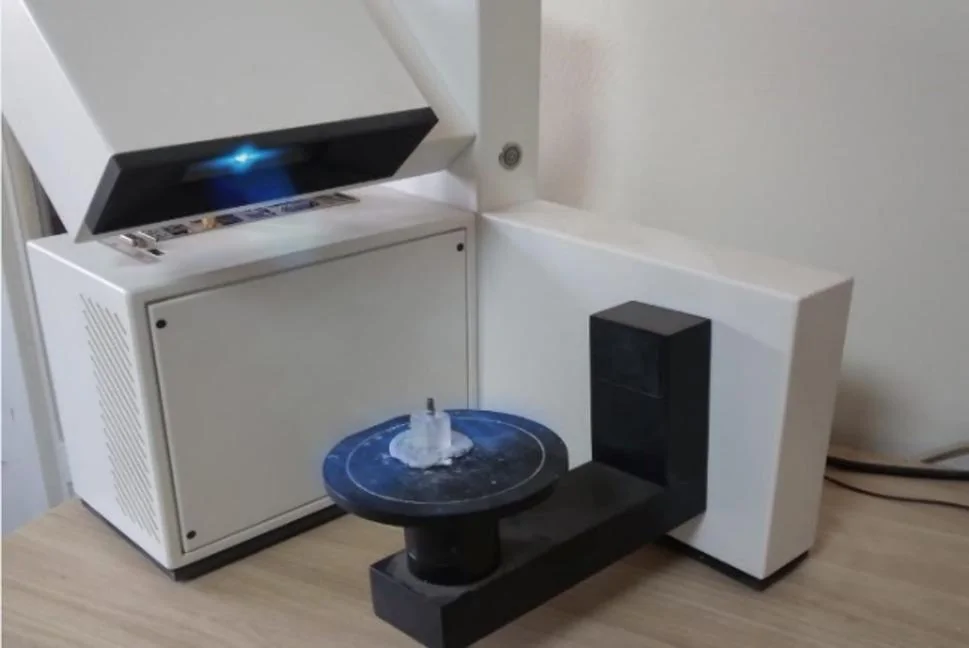
Scanning the stock abutment by a laboratory scanner. Adapted from Mostafa et al. [40]

**Fig. 8:**
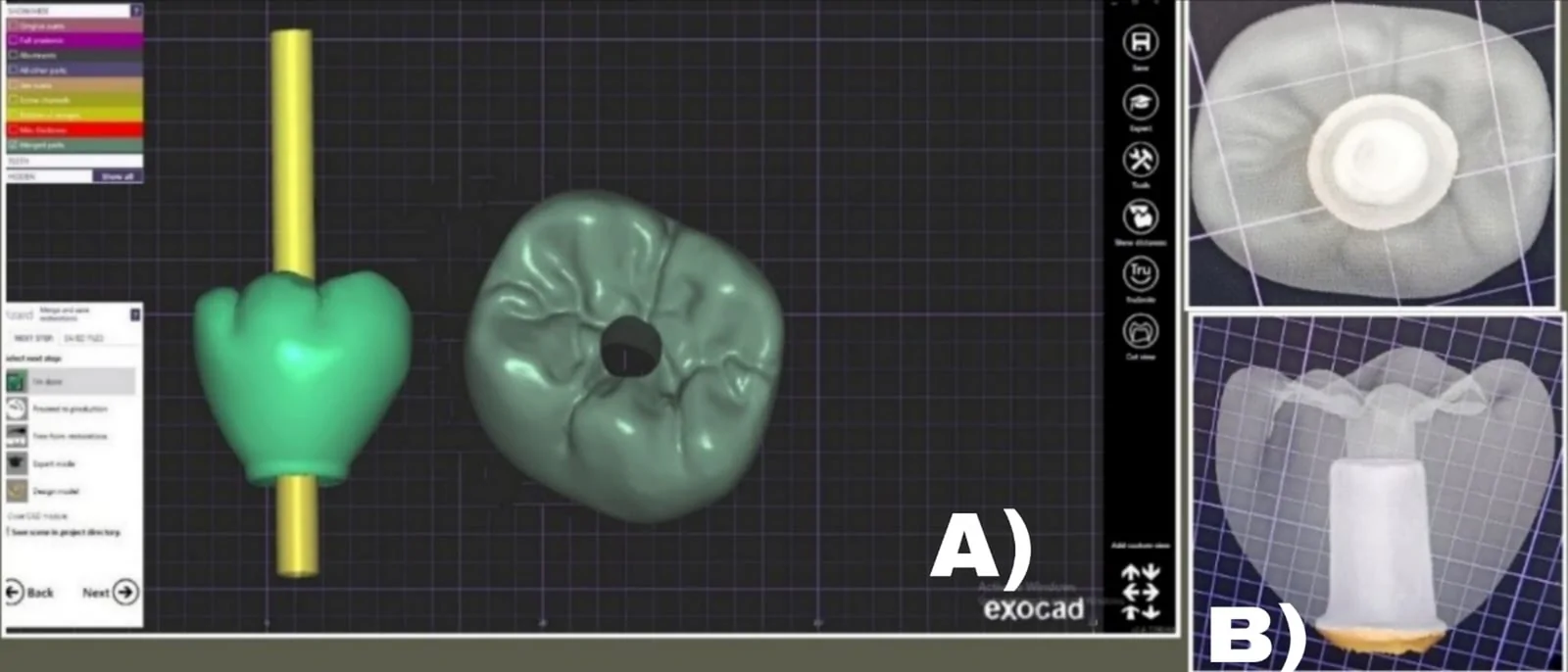
Designing of the crown using Exocad software. **A** Designing the screw access hole **B** The final shape of the designed crown

After the abutment is tightened to the implant, the abutment is scanned via a laboratory scanner ((Fig. 7), then designing of the crown was done with a screw access hole via prosthetic CAD software (Fig. 8), the crown length was normalized across all samples in both groups through the software with uniform dimensions. To ensure standardization, a single master crown design was created in Exocad with fixed parameters: 50 μm cement space, 1.5 mm occlusal thickness, and a standardized emergence profile. This master file was then duplicated for all specimens to eliminate geometry as a variable. This ensured that the crown length was consistent across all samples, zirconia crowns then milled via CAM milling machines. After milling, the crowns were sintered in a sintering furnace and then finished and polished [41].

#### Cementation of the crown

**Fig. 9:**
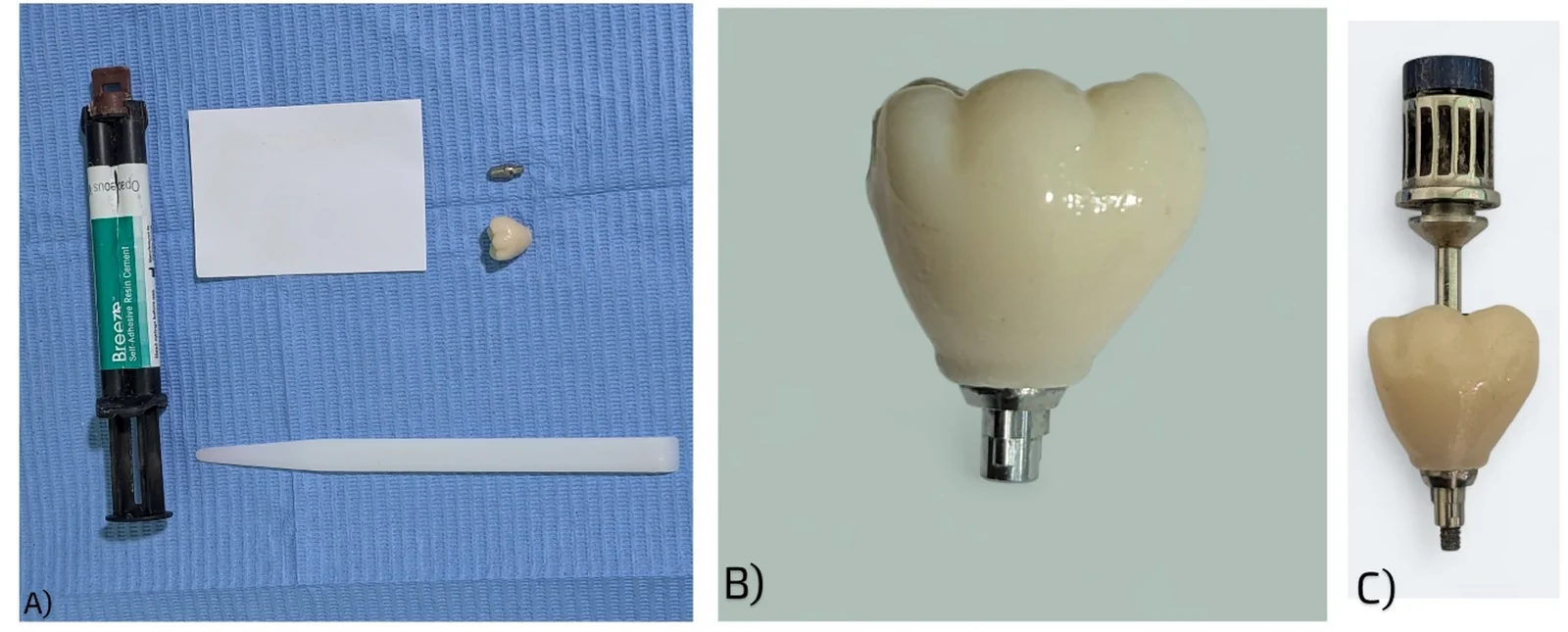
Cementation of the crown. **A** Set of the cementation protocol. **B** cemented crown to the abutment. **C** screw driver holding the screw through the screw channel in the cemented crown

Cementation of the crown to the implant was done via a hybrid approach where the crown was secured to the dental implant abutment using a dual cure resin cement [37] (Fig. 9).The crowns were cemented to the abutments extraorally to facilitate easy cement removal and minimize excess cement. To ensure standardized cement application, a thin, uniform layer of cement was applied to the abutment [42]. Each crown was initially seated onto its corresponding abutment under finger pressure then fixed to a universal testing machine and a standard load of 50 N was applied for 10 min [43], then the abutment was fixed to the implant fixture by a screw .

#### Measuring the amount of initial torque loss value

**Fig. 10:**
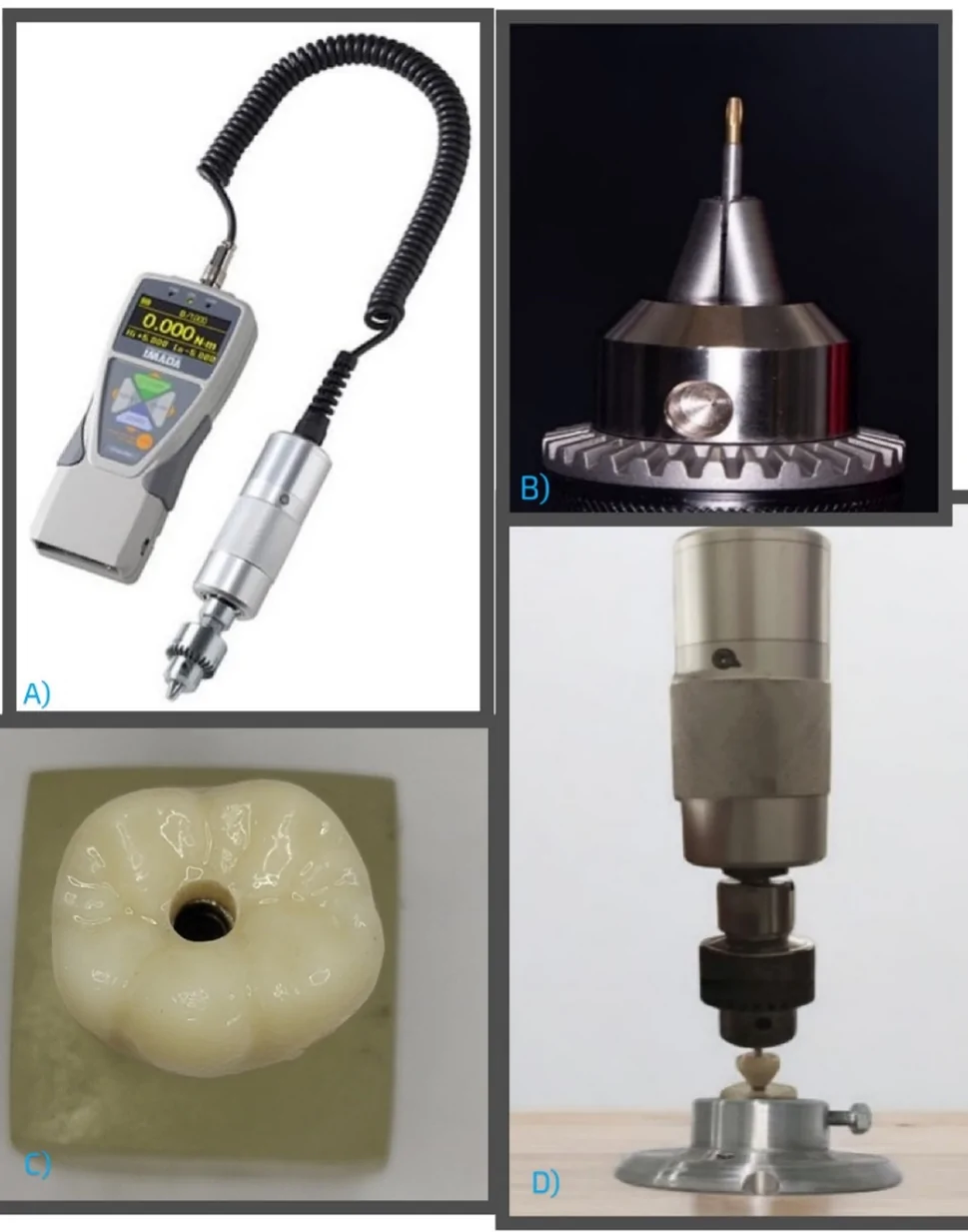
Measuring the initial torque loss value. **A** Digital torque gauge device. **B** Screw driver secured within the head of the digital torque gauge. **C** opening of the screw channel in the cemented crown **D** Measuring the initial torque loss with the torque gauge

After crown cementation to the abutment, each abutment screw was tightened to 25 Ncm via a digital torque gauge according to the manufacturer’s recommendation [44]. By holding the screw driver firmly over the screw head in the screw access hole with the long axis of the implant fixture, it was rotated clockwise. To prevent implant rotation during tightening and removal torque measurements, the epoxy resin blocks were secured in a custom-machined stainless steel holder that provided rigid stabilization. No rotational movement was observed during the application of the 25 Ncm torque. (Fig. 10)

Ten minutes after the first torque application, a torque of 25 Ncm was reapplied again with the same digital torque gauge to compensate for the settling effect or embedment relaxation of the screw and thus to assist in achieving the optimum preload [45] .Ten minutes after screw retightening, rotating the screw driver in an anticlockwise direction and recorded to determine the initial torque loss.

#### Cyclic loading application

**Fig. 11:**
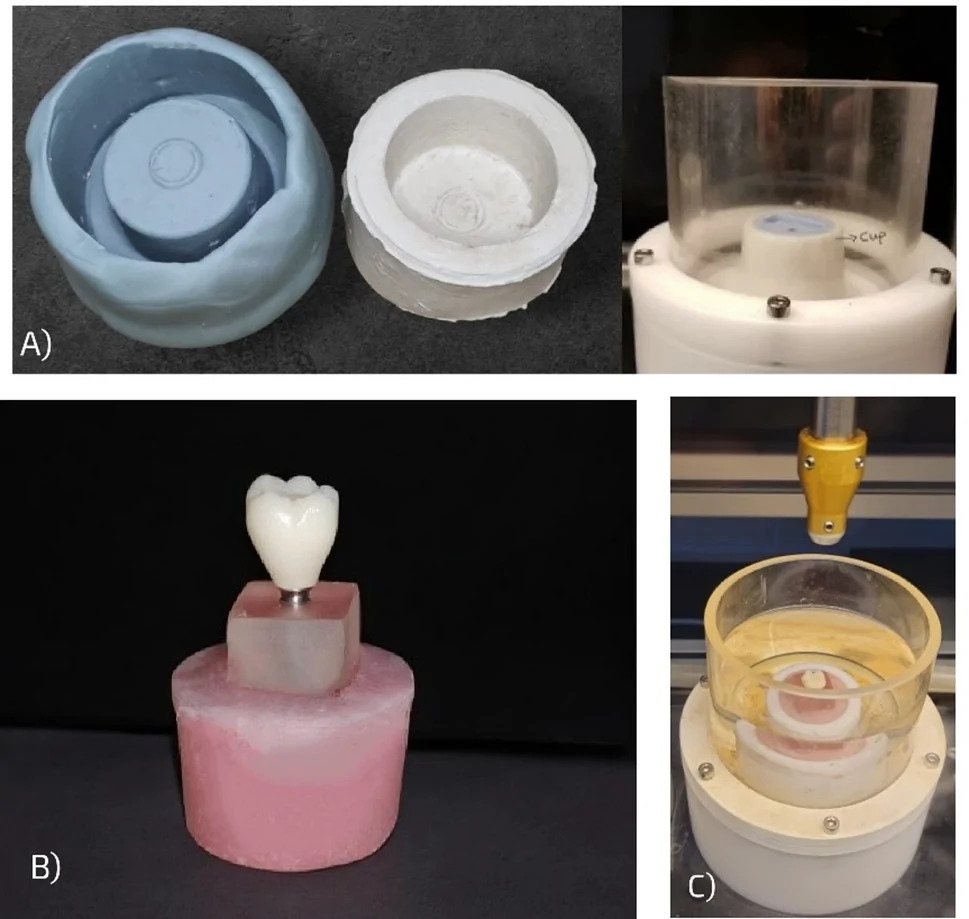
Preparation of the samples for chewing simulator device. **A** impression of the chewing simulator cup with the poured stone model. **B** Acrylic base with the block in it. **C** the acrylic base fitted in the chewing simulator cup

After measuring the initial torque loss value the screw access hole was closed by using a teflon tape over the screw then composite resin filling to avoid any interference during dynamic cyclic loading application [46]. The cyclic loading was performed using programmable logic-controlled equipment, the newly developed four-station multimodal chewing simulator CS-4, which has four chambers simulating vertical and horizontal movements, was used. Blocks preparation was based on the experimental design reported previously by Mostafa et al. [40]. Every block was prepared by taking an impression of the chewing simulator cup to make an acrylic base fit within the cup of the simulator [40] (Fig. 11) .

**Fig. 12:**
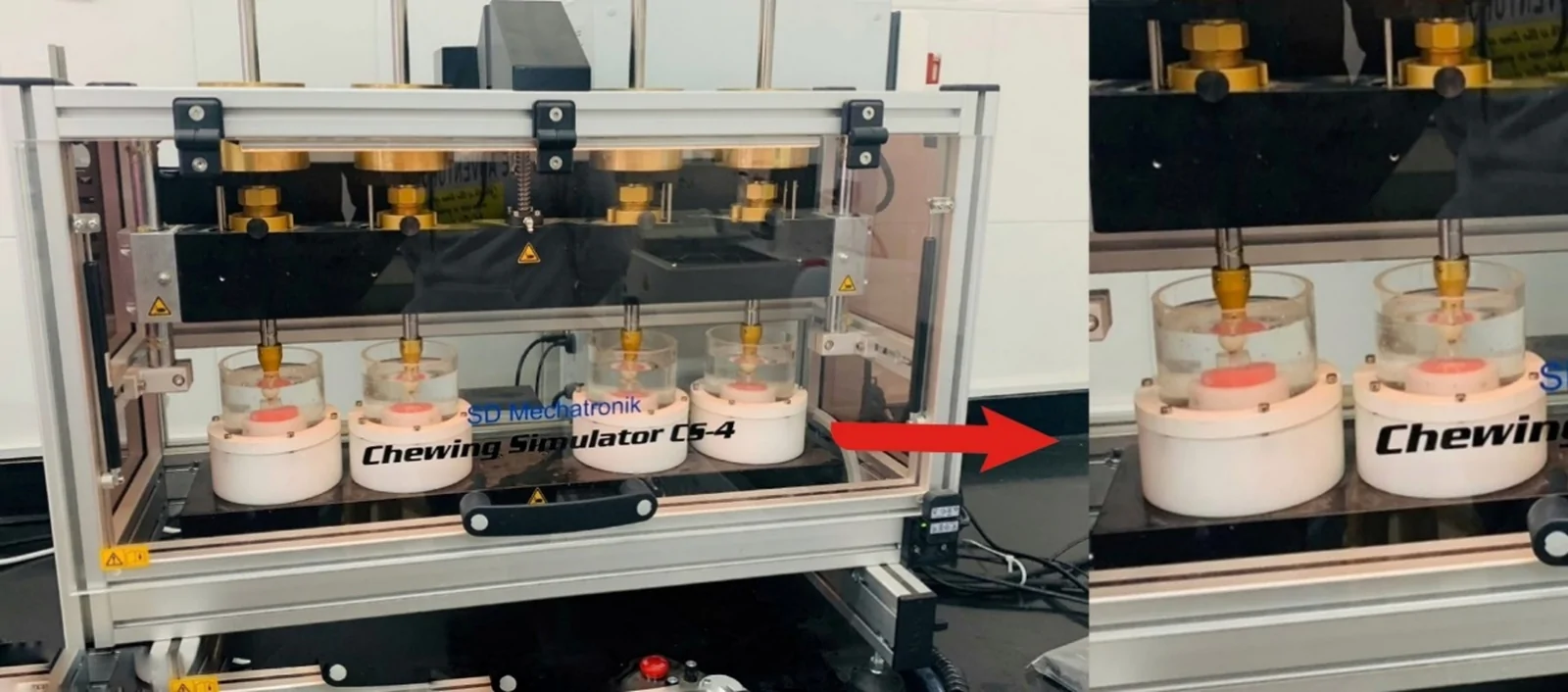
Application of dynamic cyclic loading by chewing simulator device. Adapted from Mostafa et al. [40]

The cyclic loading protocol was based on the experimental design reported previously by Mostafa et al. [40]. As dynamic cyclic loading was performed with a static ball, which was attached to the upper movable compartment of the machine to simulate the masticatory load intraorally with these parameters for 150,000 cycles of dynamic cyclic loading according to a previous study [47] (Fig. 12).

#### Post-loading removal torque value

After dynamic cyclic loading, the chewing simulator was stopped. Removing the samples from the chewing simulator device then removing the composite resin from the screw access hole, then removing the Teflon tape to expose the screw head.

Then post-load RTL value was subsequently measured via the same digital torque gauge with the same procedures used to measure the initial RTL value by rotating the screw driver within the torque gauge anticlockwise until the threaded disengagement between implant and screw occur then reading the recording on the digital torque gauge [48] .

#### The degree of the engagement between the screw and the implant was measured by incorporating the amount of torque loss by

1. Measuring the pre load torque loss values.
2. Measuring the post load torque loss values.
3. Measuring the difference between preload and post load values.

##### Calculation of the removal torque loss (RTL) ratio of the abutment screw before and after dynamic cyclic loading

Removal Torque (RT) was measured using a digital torque gauge, and the Percentage of Torque Loss (PTL) was determined using the following formula [49].

1. Removal torque loss ratio before loading (% initial RTL)

$$\:\mathrm{Initial\:RTL}=\frac{\mathrm{T}\mathrm{i}\mathrm{g}\mathrm{h}\mathrm{t}\mathrm{e}\mathrm{n}\mathrm{i}\mathrm{n}\mathrm{g}\:\mathrm{t}\mathrm{o}\mathrm{r}\mathrm{q}\mathrm{u}\mathrm{e}-\mathrm{R}\mathrm{e}\mathrm{m}\mathrm{o}\mathrm{v}\mathrm{a}\mathrm{l}\:\mathrm{t}\mathrm{o}\mathrm{r}\mathrm{q}\mathrm{u}\mathrm{e}\:\mathrm{b}\mathrm{e}\mathrm{f}\mathrm{o}\mathrm{r}\mathrm{e}\:\mathrm{l}\mathrm{o}\mathrm{a}\mathrm{d}\mathrm{i}\mathrm{n}\mathrm{g}}{\mathrm{T}\mathrm{i}\mathrm{g}\mathrm{h}\mathrm{t}\mathrm{e}\mathrm{n}\mathrm{i}\mathrm{n}\mathrm{g}\:\mathrm{t}\mathrm{o}\mathrm{r}\mathrm{q}\mathrm{u}\mathrm{e}}\mathrm{X\:100}$$

2. Torque loss ratio after loading (% postload RTL)

$$\:\mathrm{Post\:load\:RTL}=\frac{\mathrm{T}\mathrm{i}\mathrm{g}\mathrm{h}\mathrm{t}\mathrm{e}\mathrm{n}\mathrm{i}\mathrm{n}\mathrm{g}\:\mathrm{t}\mathrm{o}\mathrm{r}\mathrm{q}\mathrm{u}\mathrm{e}-\mathrm{R}\mathrm{e}\mathrm{m}\mathrm{o}\mathrm{v}\mathrm{a}\mathrm{l}\:\mathrm{t}\mathrm{o}\mathrm{r}\mathrm{q}\mathrm{u}\mathrm{e}\:\mathrm{a}\mathrm{f}\mathrm{t}\mathrm{e}\mathrm{r}\:\mathrm{l}\mathrm{o}\mathrm{a}\mathrm{d}\mathrm{i}\mathrm{n}\mathrm{g}}{\mathrm{T}\mathrm{i}\mathrm{g}\mathrm{h}\mathrm{t}\mathrm{e}\mathrm{n}\mathrm{i}\mathrm{n}\mathrm{g}\:\mathrm{t}\mathrm{o}\mathrm{r}\mathrm{q}\mathrm{u}\mathrm{e}}\mathrm{X\:100}$$

3. Removal torque loss ratio before and after loading (% difference between initial and postload RTL)

$$\:\mathrm{Difference\:in\:RTL}=\frac{\mathrm{R}\mathrm{e}\mathrm{m}\mathrm{o}\mathrm{v}\mathrm{a}\mathrm{l}\:\mathrm{t}\mathrm{o}\mathrm{r}\mathrm{q}\mathrm{u}\mathrm{e}\:\mathrm{b}\mathrm{e}\mathrm{f}\mathrm{o}\mathrm{r}\mathrm{e}\:\mathrm{l}\mathrm{o}\mathrm{a}\mathrm{d}\mathrm{i}\mathrm{n}\mathrm{g}-\mathrm{R}\mathrm{e}\mathrm{m}\mathrm{o}\mathrm{v}\mathrm{a}\mathrm{l}\:\mathrm{t}\mathrm{o}\mathrm{r}\mathrm{q}\mathrm{u}\mathrm{e}\:\mathrm{a}\mathrm{f}\mathrm{t}\mathrm{e}\mathrm{r}\:\mathrm{l}\mathrm{o}\mathrm{a}\mathrm{d}\mathrm{i}\mathrm{n}\mathrm{g}}{\mathrm{R}\mathrm{e}\mathrm{m}\mathrm{o}\mathrm{v}\mathrm{a}\mathrm{l}\:\mathrm{t}\mathrm{o}\mathrm{r}\mathrm{q}\mathrm{u}\mathrm{e}\:\mathrm{b}\mathrm{e}\mathrm{f}\mathrm{o}\mathrm{r}\mathrm{e}\:\mathrm{l}\mathrm{o}\mathrm{a}\mathrm{d}\mathrm{i}\mathrm{n}\mathrm{g}}\mathrm{X\:100}$$

## Results

**Fig. 13:**
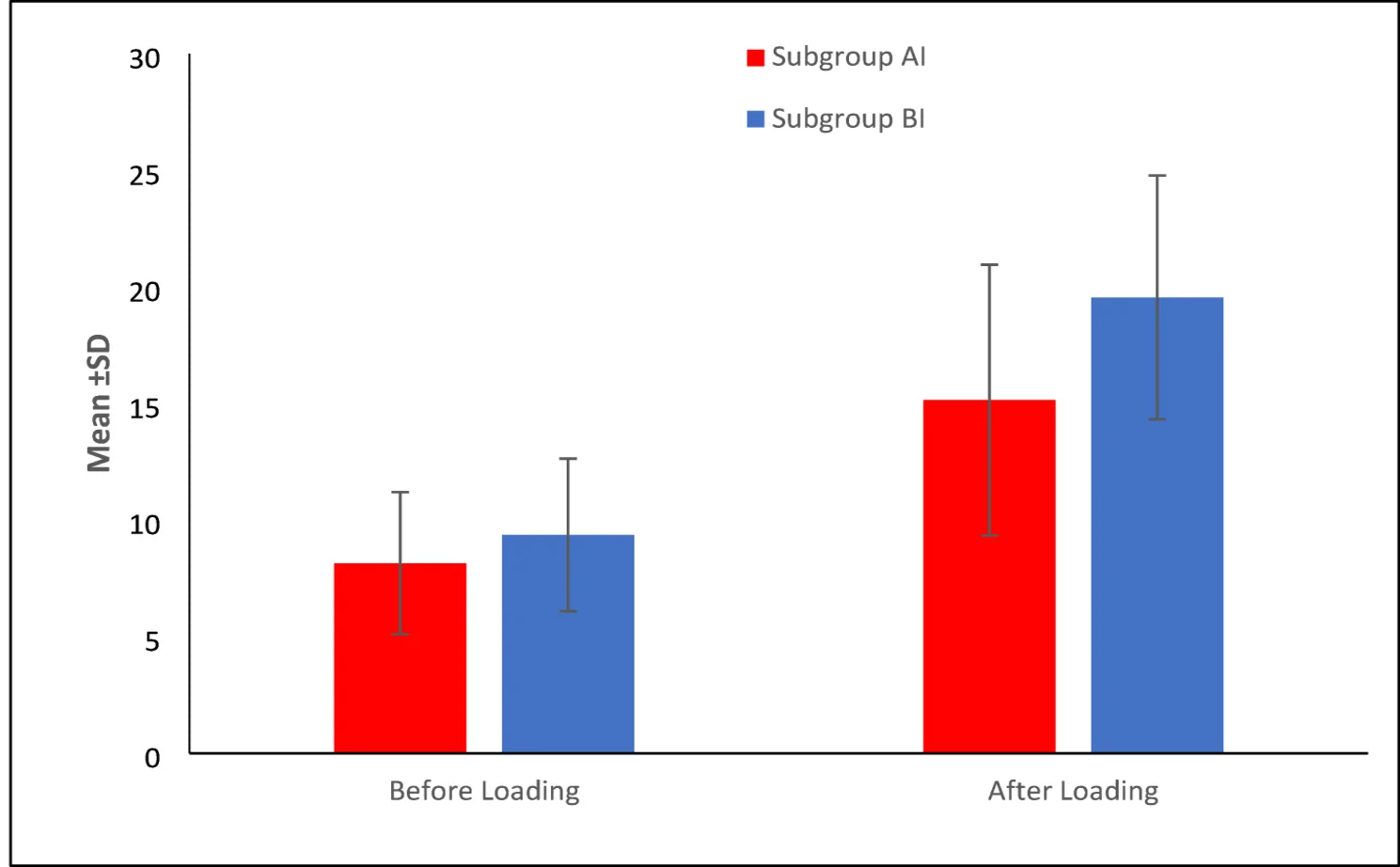
Showing removal torque loss ratio for both subgroups (A&B) in Group (I) before loading and after Loading

**Fig. 14:**
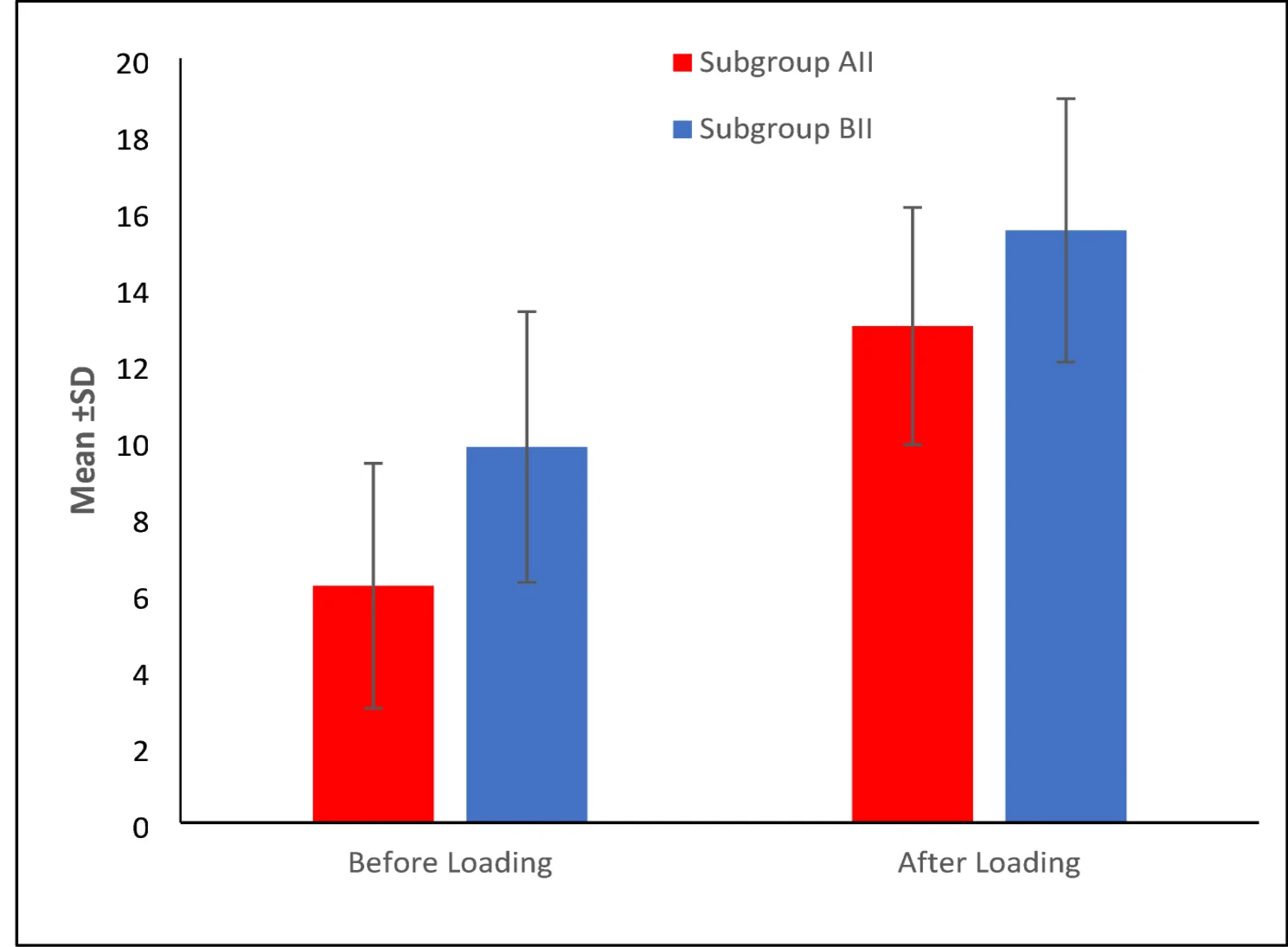
Showing removal torque loss ratio for both subgroups (A&B) in Group (II) before and after Loading

**Fig. 15:**
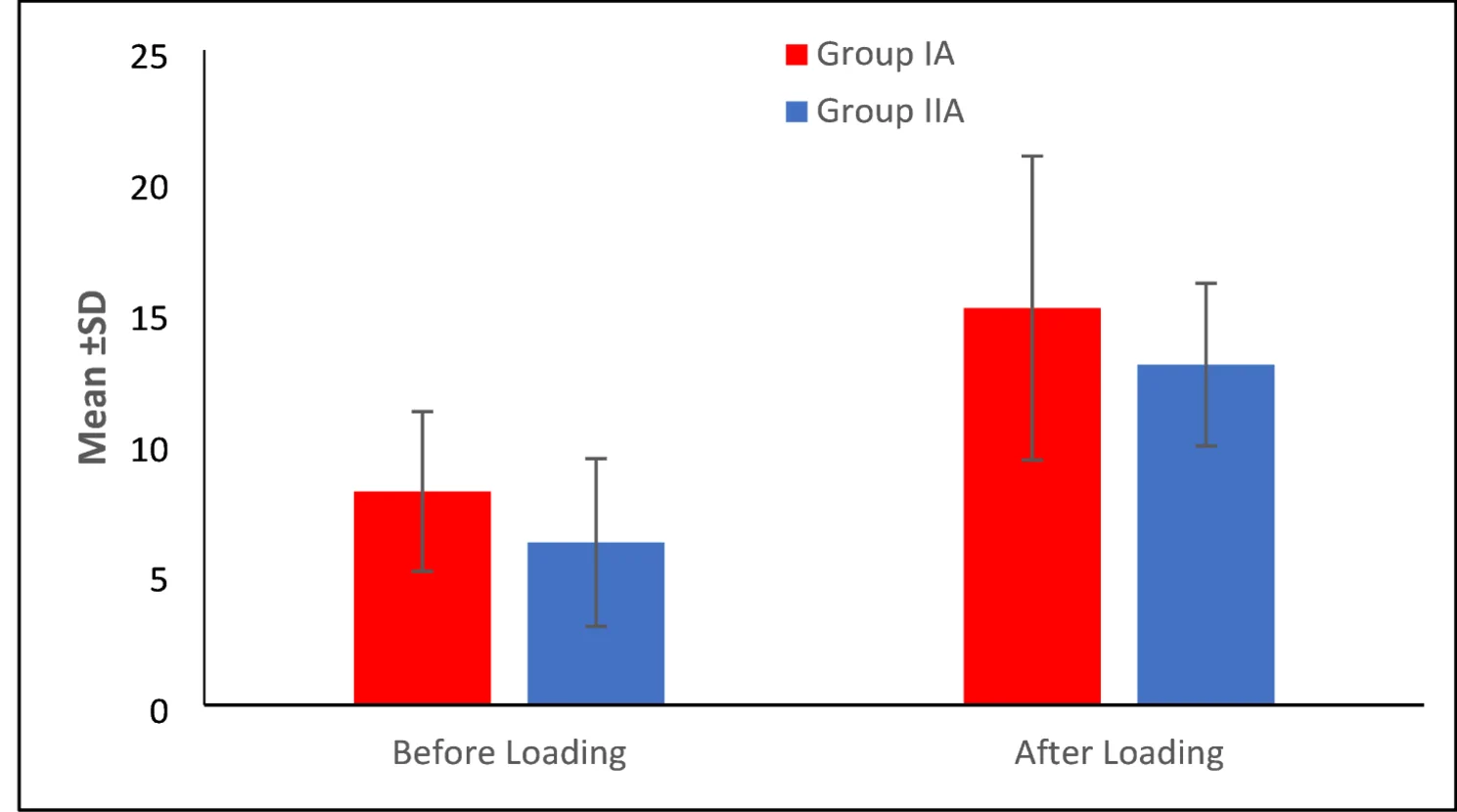
Showing the removal torque loss results at before and after loading for subgroup A in both group (I&II)

**Fig. 16:**
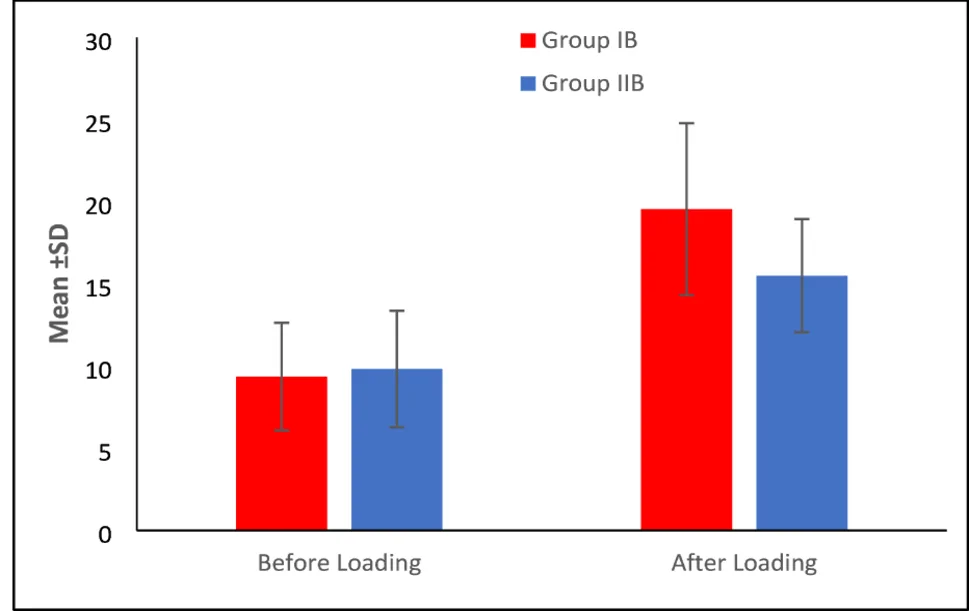
Showing the removal torque loss results at before and after loading for subgroup B in both group (I&II)

The data were analyzed using IBM SPSS Statistics (v26.0). Descriptive statistics (Mean ± SD) were calculated for all groups. A Two-Way Mixed ANOVA was conducted to evaluate the effect of time (Pre-loading vs. Post-loading) and the interaction between the groups. Additionally, a Factorial ANOVA was utilized to determine the influence of connection geometry and fabrication technique on the torque loss ratio. The level of significance was set at $P < 0.05$. Post-hoc comparisons were performed using the Bonferroni correction to identify specific differences between subgroups (Tables 1, 2, 3 and 4) (Figs. 13, 14, 15 and 16).

**Table 1:** Showing removal torque loss ratio in Group I (Internal trilobe connection) for both subgroups (Stock & Custom-made abutment) before loading and after Loading

Removal Torque Loss Ratio		Subgroup						Independent T-Test	
		Subgroup AI			Subgroup BI			t	P-value
Before Loading	Range	3.2	-	14.2	4.4	-	15.6	-1.084	0.287
	Mean ±SD	8.150	±	3.048	9.363	±	3.276		
After Loading	Range	4.4	-	27.6	11.2	-	30.2	-2.254	0.032*
	Mean ±SD	15.150	±	5.804	19.551	±	5.227		
Paired Differences	Mean ±SD	-7.000	±	6.367	-10.188	±	6.920		
Paired Test	t	-4.398			-5.889				
	P-value	0.001*			<0.001*				

There is a significant at P-value< 0.05 (*), and highly significant at P-value< 0.001 (**)

**Table 2:** Showing removal torque loss ratio in Group II (Internal Hexagonal connection) for both subgroups (Stock & Custom-made abutment) before loading and after Loading

Removal Torque Loss Ratio		Subgroup						Independent T-Test	
		Subgroup AII			Subgroup BII			t	*P*-value
Before Loading	Range	2	-	13.2	4.1	-	18.4	-3.041	0.005*
	Mean ±SD	6.200	±	3.203	9.831	±	3.542		
After Loading	Range	8.8	-	18.4	10.4	-	21.2	-2.162	0.039*
	Mean ±SD	12.994	±	3.105	15.500	±	3.445		
Paired Differences	Mean ±SD	-6.794	±	3.642	-5.669	±	4.711		
Paired Test	t	-7.461			-4.814				
	*P*-value	<0.001*			<0.001*				

There is a significant at *P*-value< 0.05 (*), and highly significant at *P*-value< 0.001 (**)

**Table 3:** Showing removal torque loss ratio in Sungroup A (Stock Abutment) for both Ggroups (Internal Trilobe & Hexagonal connection) before loading and after Loading

Removal Torque Loss Ratio		Group						Independent T-Test	
		Group IA			Group IIA			t	P-value
Before Loading	Range	3.2	-	14.2	2	-	13.2	1.764	0.088
	Mean ±SD	8.150	±	3.048	6.200	±	3.203		
After Loading	Range	4.4	-	27.6	8.8	-	18.4	1.310	0.200
	Mean ±SD	15.150	±	5.804	12.994	±	3.105		
Paired Differences	Mean ±SD	-7.000	±	6.367	-6.794	±	3.642		
Paired Test	t	-4.398			-7.461				
	P-value	0.001*			<0.001*				

There is a significant at *P*-value< 0.05 (*), and highly significant at *P*-value< 0.001 (**)

**Table 4:** Showing removal torque loss ratio in Sungroup B (Custom-made Abutment) for both Ggroups (Internal Trilobe & Hexagonal connection) before loading and after Loading

Removal Torque Loss Ratio		Group						Independent T-Test	
		Group IB			Group IIB			t	P-value
Before Loading	Range	4.4	-	15.6	4.1	-	18.4	-0.389	0.700
	Mean ±SD	9.363	±	3.276	9.831	±	3.542		
After Loading	Range	11.2	-	30.2	10.4	-	21.2	2.588	0.015*
	Mean ±SD	19.551	±	5.227	15.500	±	3.445		
Paired Differences	Mean ±SD	-10.188	±	6.920	-5.669	±	4.711		
Paired Test	t	-5.889			-4.814				
	P-value	<0.001*			<0.001*				

There is a significant at *P*-value< 0.05 (*), and highly significant at *P*-value< 0.001 (**)

1. Intra-group Comparison: Before vs. After Loading

Across all experimental subgroups, there was a statistically significant increase in the Removal Torque Loss (RTL) Ratio following the loading phase.

- Group I (Subgroups IA & IB): In Subgroup IA, the mean RTL ratio increased from 8.150 ± 3.048% before loading to 15.150 ± 5.804% after loading (P=0.001). Similarly, Subgroup IB showed a significant increase from 9.363 ± 3.276% to 19.551 ± 5.227% (P<0.001) as shown in table 1.
- Group II (Subgroups IIA & IIB): Subgroup IIA rose from 6.200 ± 3.203% to 12.994 ± 3.105% (P<0.001) , while Subgroup IIB increased from 9.831 ± 3.542% to 15.500 ± 3.445% (P<0.001) as shown in table 2.
- Overall Trend: When considering the entire sample, the mean RTL ratio for all groups combined significantly jumped from 8.386 ± 3.493% to 15.799 ± 5.035% after loading (P<0.001).

2. Inter-group Comparison: Group I vs. Group II

When comparing the two main groups (Group I vs. Group II), the differences were generally not statistically significant for the combined mean values, though specific interactions were noted.

- The overall mean RTL ratio for Group I (13.053 ± 6.379%) was slightly higher than Group II (11.131 ± 4.784%), but this difference did not reach statistical significance ($*P* = 0.056$).
- When examining the Before and After Loading combined data specifically, the difference between Group I (8.541%) and Group II (8.047%) remained non-significant ($*P* = 0.629$).

3. Subgroup Analysis (Subgroup A vs. Subgroup B)

**Fig. 17:**
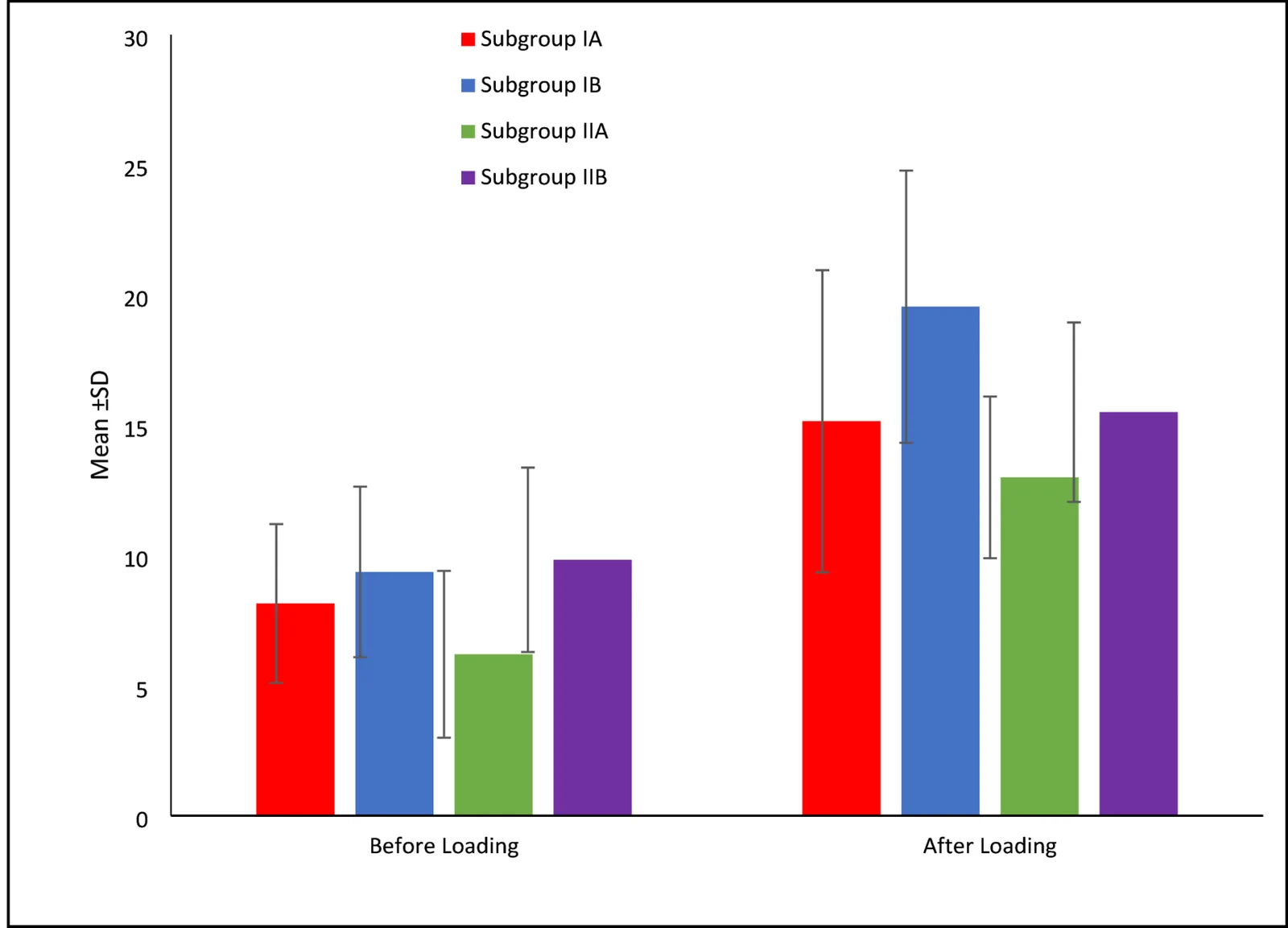
Showing removal torque loss ratio for both Group (I&II) with the subgroups before and after Loading

**Fig. 18:**
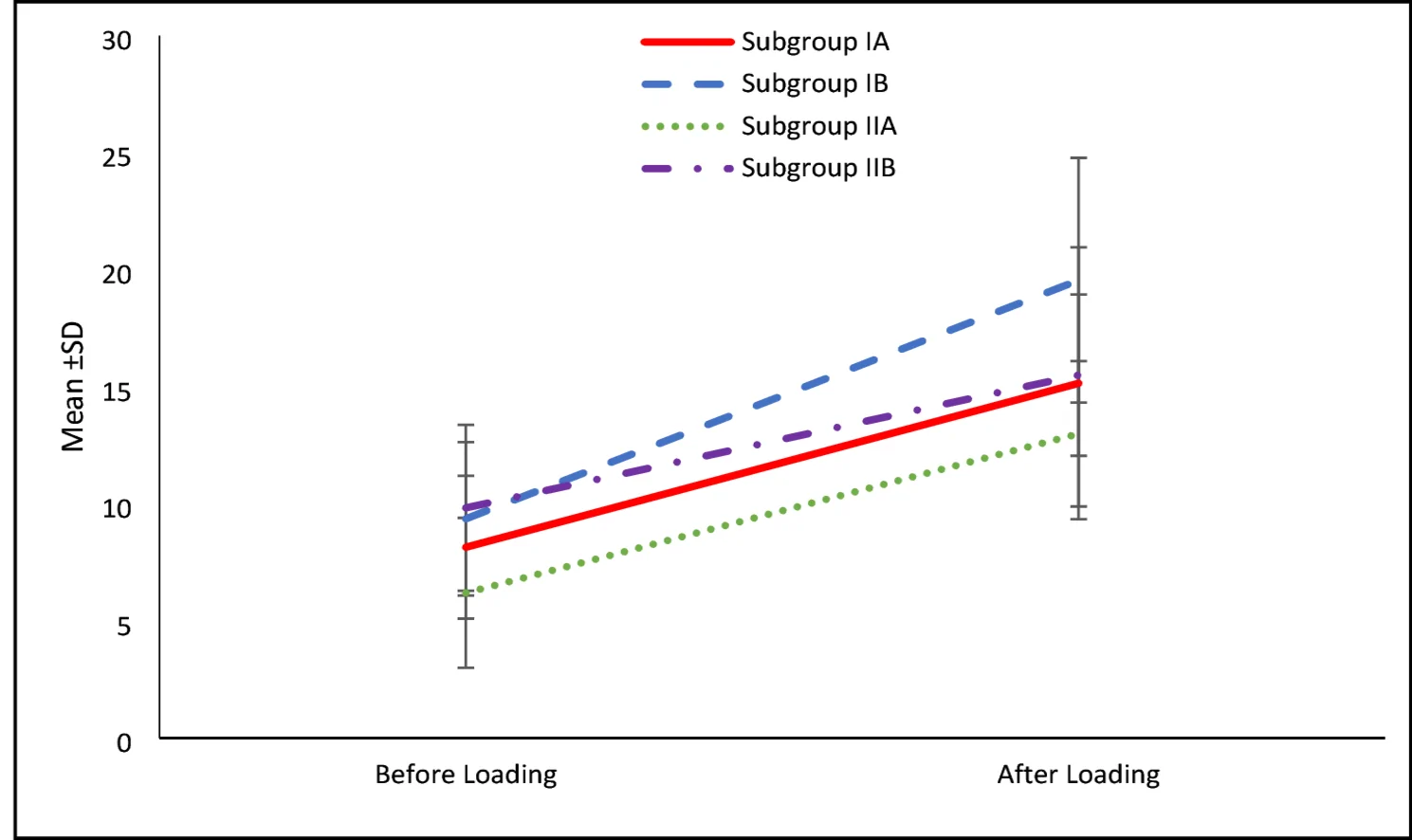
Showing removal torque loss ratio for both Group (I&II) with the subgroups before and after Loading.(Linear Figure)

Subgroup B consistently demonstrated higher torque loss ratios compared to Subgroup A (Table 5) (Figs. 17 and 18).

- Comparison within Group I: After loading, Subgroup IB (19.551%) had a significantly higher RTL ratio than Subgroup IA (15.150%) ($P = 0.032$).
- Comparison within Group II: Subgroup BII showed significantly higher RTL ratios than Subgroup AII both before loading ($P = 0.005$) and after loading ($P = 0.039$).
- Total Subgroup Comparison: Looking at the total data, Subgroup B (13.561 ± 5.740%) showed a significantly higher RTL ratio than Subgroup A (10.623 ± 5.303%) with $P = 0.003$.

**Table 5:** Showing removal torque loss ratio for both Group (Trilobe & Hexagonal) with the subgroups before and after Loading using Multiple Comparison (Tukey’s Post-Hoc Test)

Removal Torque Loss Ratio		Subgroups												One Way ANOVA		
		Subgroup IA			Subgroup IB			Subgroup IIA			Subgroup IIB			F		*P*-value
BeforeLoading	Range	3.2	-	14.2	4.4	-	15.6	2	-	13.2	4.1	-	18.4	3.923		0.013*
	Mean ±SD	8.150	±	3.048	9.363	±	3.276	6.200	±	3.203	9.831	±	3.542			
After Loading	Range	4.4	-	27.6	11.2	-	30.2	8.8	-	18.4	10.4	-	21.2	5.806		0.001*
	Mean ±SD	15.150	±	5.804	19.551	±	5.227	12.994	±	3.105	15.500	±	3.445			
Paired Differences	Mean ±SD	-7.000	±	6.367	-10.188	±	6.920	-6.794	±	3.642	-5.669	±	4.711			
Paired Test	t	-4.398			-5.889			-7.461			-4.814					
	*P*-value	0.001*			<0.001*			<0.001*			<0.001*					

TUKEY'S Test																
	IA&IB		IA&IIA			IA&IIB			IB&IIA			IB&IIB			IIA&IIB	
Before	0.722		0.340			0.472			0.040*			0.977			0.014*	
After	0.039*		0.540			0.996			0.001*			0.067			0.409	

There is a significant at *P*-value< 0.05 (*), and highly significant at *P*-value< 0.001 (**)

4. The One-Way ANOVA revealed significant differences between specific subgroups (Table 6)

• Before Loading: Significant differences were found between IB & IIA ($*P* = 0.040$) and IIA & IIB ($*P* = 0.014$).

• After Loading: Significant differences were found between IA & IB ($*P* = 0.039$) and IB & IIA ($*P* = 0.001$).

**Table 6:** Showing removal torque loss ratio for both Groups using Factorial ANOVA

Tests of Between-Subjects Effects					
Dependent Variable:	Removal Torque Loss Ratio				
Source	Type III Sum of Squares	df	Mean Square	F	P-value
Corrected Model	2243.620^a^	7	320.517	20.457	<0.001*
Intercept	18716.530	1	18716.530	1194.559	<0.001*
Group	118.215	1	118.215	7.545	0.007*
Subgroup	276.154	1	276.154	17.625	<0.001*
Time	1758.319	1	1758.319	112.222	<0.001*
Group * Subgroup	0.550	1	0.550	0.035	0.852
Group * Time	44.663	1	44.663	2.851	0.094
Subgroup * Time	8.513	1	8.513	0.543	0.462
Group * Subgroup * Time	37.206	1	37.206	2.375	0.126
Error	1880.178	120	15.668		
Total	22840.328	128			
Corrected Total	4123.798	127

**Fig. 19:**
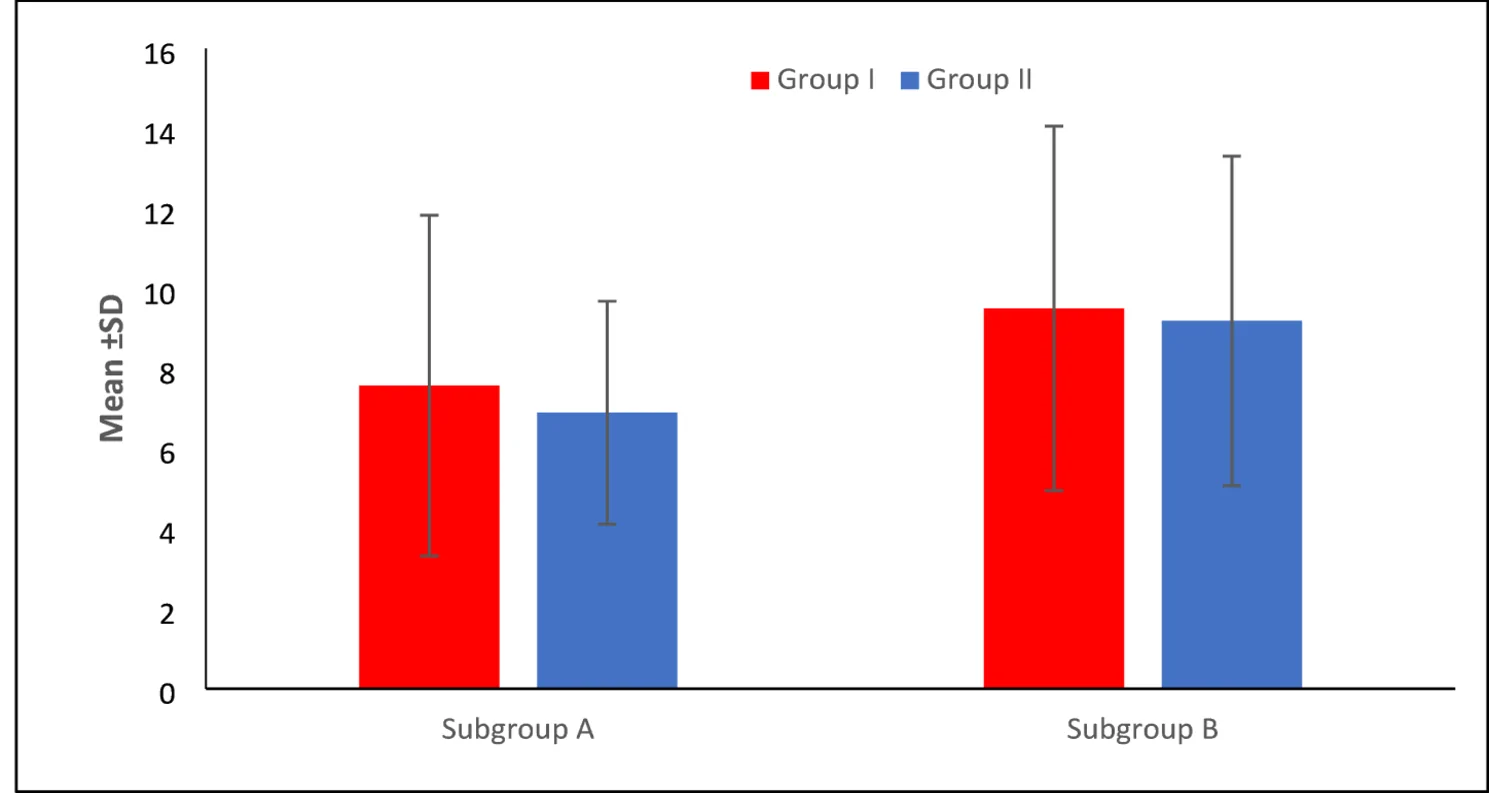
Showing the comparison between the studied groups at removal torque loss difference between before and after loading

5. Factors Affecting Removal Torque Loss (Factorial ANOVA) (Table 7, (Figs. 19and20. (Table 8, Fig. 21) and Table 9.

The Tests of Between-Subjects Effects identified the primary drivers of torque loss:

- Group ($*P* = 0.007$), Subgroup ($*P* < 0.001$), and Time ($*P* < 0.001$) all had a highly significant independent impact on the RTL ratio.
- However, there were no significant interactions between these factors (e.g., Group * Subgroup or Group * Time), suggesting that the effect of loading (Time) or the material (Subgroup) acted independently of the main group classification.
- For the combined “Before and After” metric, only the Subgroup remained a significant factor ($*P* = 0.038$).

**Table 7:** Showing the comparison between the studied groups (Internal Trilobe & Hexagonal ) at removal torque loss difference between before and after loading

Removal Torque Loss Ratio Before and After Loading		Group						Independent T-Test	
		Group I(Internal Trilobe)			Group II(Internal Hexagonal)			t	P-value
Subgroup A(Stock Abutment)	Range	2	-	16	3.2	-	12.4	0.531	0.599
	Mean ±SD	7.575	±	4.257	6.900	±	2.785		
Subgroup B(Custom-Made abutment)	Range	2.8	-	18.1	2.8	-	16	0.204	0.840
	Mean ±SD	9.506	±	4.554	9.194	±	4.116		
Independent T-Test	t	-1.239			-1.846				
	P-value	0.225			0.075				

There is a significant at P-value< 0.05 (*), and highly significant at P-value< 0.001 (**).

**Fig. 20:**
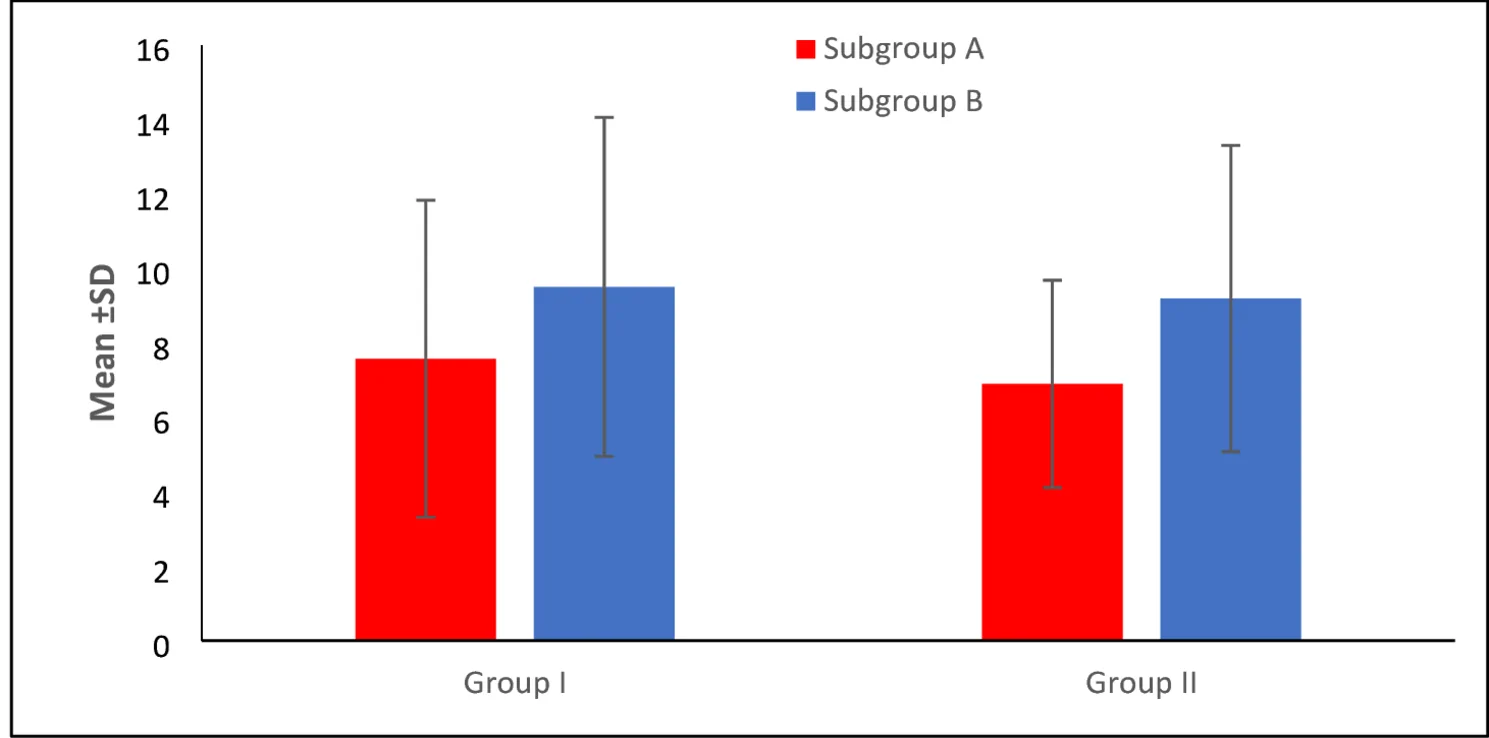
Show the comparison between the studied groups at removal torque loss difference between before and after loading

**Table 8:** Showing the comparison between the studied groups at removal torque loss difference between before and after loading

Removal Torque Loss Ratio Before and After Loading	Subgroups												One Way ANOVA	
	Subgroup IA			Subgroup IB			Subgroup IIA			Subgroup IIB			F	P-value
Range	2	-	16	2.8	-	18.1	3.2	-	12.4	2.8	-	16	1.591	0.201
Mean ±SD	7.575	±	4.257	9.506	±	4.554	6.900	±	2.785	9.194	±	4.116		

There is a significant at *P*-value< 0.05 (*), and highly significant at *P*-value< 0.001 (**)

**Fig. 21:**
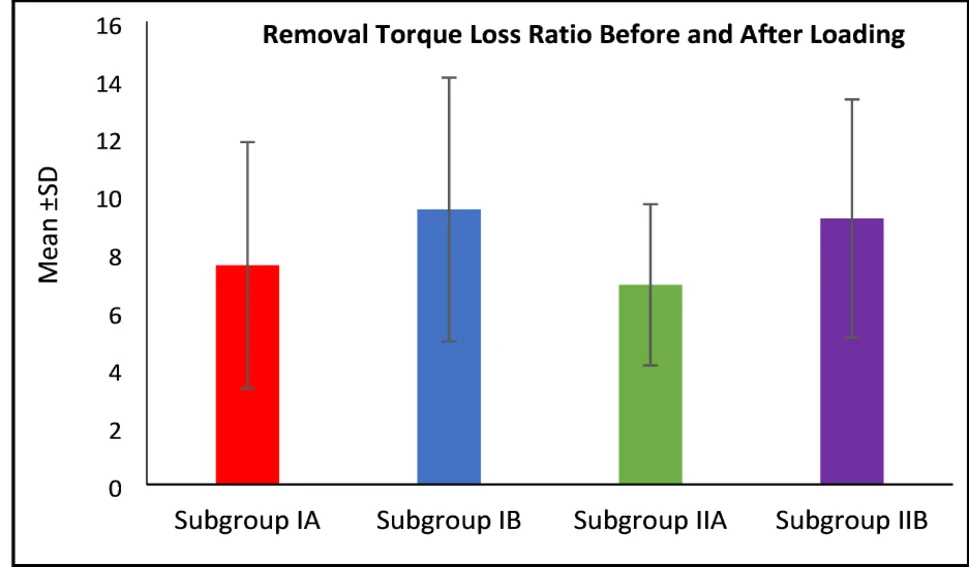
Show the comparison between the studied groups at removal torque loss difference between before and after loading

**Table 9:** Showing difference in removal torque loss ratio for both Groups using Factorial ANOVA

Tests of Between-Subjects Effects					
Dependent Variable:	Removal Torque Loss Ratio Before and After Loading				
Source	Type III Sum of Squares	df	Mean Square	F	P-value
Corrected Model	75.829^a^	3	25.276	1.591	0.201
Intercept	4402.323	1	4402.323	277.047	<0.001*
Group	3.901	1	3.901	0.245	0.622
Subgroup	71.402	1	71.402	4.494	0.038*
Group * Subgroup	0.526	1	0.526	0.033	0.856
Error	953.409	60	15.890		
Total	5431.560	64			
Corrected Total	1029.238	63			

There is a significant at *P*-value< 0.05 (*), and highly significant at *P*-value< 0.001 (**).

1. Comparative Analysis by Group and Subgroup

An Independent T-test was utilized to compare the RTL ratios between Group I and Group II within their respective subgroups.

- Subgroup A: Group I (7.575 ± 4.257) showed a slightly higher mean RTL ratio than Group II (6.900 ± 2.785), but this difference was not statistically significant ($*P* = 0.599$).
- Subgroup B: Similarly, no significant difference was found between Group I (9.506 ± 4.554) and Group II (9.194 ± 4.116) ($*P* = 0.840$).
- Overall Group Comparison: When pooling the data, the total mean RTL for Group I (8.541 ± 4.446) and Group II (8.047 ± 3.648) showed no statistically significant difference ($*P* = 0.629$).

2. Influence of Subgroups (A vs. B)

A significant finding emerged when comparing the experimental subgroups regardless of the primary group.

- Subgroup Effect: Subgroup B exhibited a significantly higher mean RTL ratio (9.350 ± 4.273) compared to Subgroup A (7.238 ± 3.555).
- Statistical Significance: This difference was confirmed by an Independent T-test ($P = 0.035$) and corroborated by the Tests of Between-Subjects Effects ($P = 0.038$).

3. Interaction and Multivariate Analysis

To understand the relationship between the variables, a Two-Way ANOVA (Tests of Between-Subjects Effects) was performed.

- Main Effects: The analysis confirmed that the “Subgroup” factor had a significant impact on the RTL ratio ($F = 4.494, *P* = 0.038$), whereas the “Group” factor did not ($F = 0.245, *P* = 0.622$).
- Interaction Effect: There was no significant interaction between Group and Subgroup ($*P* = 0.856$), suggesting that the effect of the subgroup treatment was consistent across both Group I and Group II.
- Overall Model: The One-Way ANOVA comparing all four individual cohorts (IA, IB, IIA, IIB) resulted in a $P$-value of $0.201$, indicating that while specific sub-factors are influential, the combined variance across all four specific categories did not reach significance.

## Discussion

To ensure high scientific rigor, this study utilized epoxy resin as a bone analogue (E ~ 20 GPa) to accurately simulate the elastic modulus of human mandibular bone [50]. As the implant is in the vertical position in the resin, it simulates real positioning in the lower mandible [51].

A standardized protocol was employed using uniform implant dimensions (13 mm x 4.2 mm) and a resin guide to eliminate positional variables that could influence screw stability. By utilizing internal implant-abutment connections (IAC), we aimed to optimize stress distribution at the marginal bone level, a factor known to mitigate screw loosening [39, 52, 53] .

Internal trilobe and internal hex connection were used in this study because of the oblique shape of the fixture to the abutment connecting surface which enhances the mechanical stability by the friction and wedging effect [54] .

When compared to the traditional casted frames, the precision of the frameworks has increased as a result of the use of CAD/CAM in dentistry [55], The capability of CAD/CAM systems to manufacture prosthetic components (abutment) with improved fitting accuracy in comparison to that achieved with conventional manufacturing procedures using press or casting techniques should be their primary characteristic [56].

Digital implant impressions for a single implant can be scanned with high predictability, as has been shown in several studies and case reports [57]. However, when using monolithic restorations, a seamless digital workflow is achievable, enabling virtual design and manufacturing of abutments and crowns [58]. In this study, monolithic zirconia was utilized for all crown restorations to standardize the experimental conditions. Zirconia’s high modulus of elasticity ensures that occlusal forces are efficiently transferred through the abutment to the screw joint, making it an ideal ‘worst-case scenario’ material for testing screw stability. However, it is important to note that restorative materials with lower stiffness, such as metal-ceramic or resin-matrix ceramics, may provide a damping effect that could alter the magnitude of torque loss. Consequently, the findings of this study should be interpreted within the context of rigid monolithic restorations, and future research is warranted to evaluate how different restorative materials influence the biomechanical behavior of these connections [59].

In our study, To ensure standardization and accuracy, abutment screws were tightened to the manufacturer-recommended torque using a digital gauge [44].Optimal torque application is crucial for successful implant restoration, as it creates a preload force within the screw [60]. Too little torque may allow separation of the joint and result in screw fatigue, loosening, and failure, while too much torque may strip the screw threads [61]. To compensate for Detorque Values due to screw settling, screws were retightened to the same torque after a 10-minute interval, ensuring optimal preloading [62]. The settling effect or embedment relaxation is the result of no surface being completely smooth [63]. Regardless of how precisely the surface of the implant was machined, during production, it was somewhat rough. No two surfaces are in close contact with one another between the screw threads and the implant threads because of the microroughness on the surface of the implant components [64] .

All samples underwent dynamic cyclic loading (150,000 cycles) using a chewing simulator, which mimics clinical loading conditions equivalent to one year of clinical use, as validated by Martin Rosentritt et al. [65] who stated that after the calculation with exponential decay gave a correlation between in vivo data and in vitro simulation. Using 50 N, a total of (750,000) of dynamic loading was necessary for simulating 5 years of oral service.

According to Kim et al. [38], the ratio of removal torque before loading and tightening torque can be an indicator of how screw disengagement takes place before loading. The ratio of removal torque after loading and tightening torque can be an indicator of how much loosening occurs after loading. The ratio of removal torque between before and after loading can be an indicator of the degree of loosening caused by the dynamic load.

Our findings demonstrated a baseline removal torque loss (RTL) in both hexagonal and trilobe groups prior to loading, with no significant difference between connection types (*p* > 0.05). This result aligns with Varvara et al. [66] and Winkler et [67] al. and Useng Yi et al. [68], This confirms a critical biomechanical principle: early torque loss is primarily driven by “embedment relaxation” or the settling effect. Even with precise machining, microroughness at the interface prevents perfect apposition. Our study reinforces the clinical necessity of the “10-minute retightening” protocol to maximize preload and compensate for this initial 2%–10% decrease in clamping force [23, 69]. Our results compatible with other studies reported that the untightening torque was almost 2 to 3 Ncm less than the tightening torque of the prosthesis-retaining screws [70].

In our study, a significant increase in torque loss ratio was observed in both groups I and II after loading, consistent with EMAN et al. [45], this phenomenon is attributed to surface settling, screw deformation, axial displacement of the abutment, which allows the tension at the screw thread interface to be reduced, the surface sinking phenomenon of the implant system, joint micromovement, and preload loss within the implant system [69] .

The result of difference in RTL before and after loading can described by Bickford et al. [71], who stated that screw loosening occur in two stages initially, external forces causes sliding between the threads, partially relieving the stretching of the screw and reducing preload. At this stage, the higher the preload the greater will be the resistance to loosening. The second stage is attained by gradual reduction of preload below a critical level, in which external forces cause the turning of the screw in an anti-clockwise direction, and it loses its function.

While removal torque is a widely accepted method for evaluating the mechanical integrity of the implant-abutment connection, its interpretation has inherent limitations. It serves as an indirect indicator of joint stability rather than a direct measurement of tensile preload. The values recorded are influenced by the settling effect, a phenomenon where microscopic surface irregularities are flattened under load and changes in the coefficient of friction at the screw-abutment interface. Therefore, while our results demonstrate significant relative differences between stock and custom-made components, they do not account for the absolute preload forces existing within the screw joint.

In our study the mean of RTL was grater in CAD/CAM abutments than stock abutments and the difference was statistically significant and this finding is in agreement with Useng Yi et al. [72]., who concluded that CAD/CAM abutments presented a significantly greater removal torque reduction than prefabricated abutments. Also this is in agreement with Soo-Yeon Shin et al. [73] who found that after a 10^5^ cycles of dynamic load, CAD/CAM custom abutment had an effect on screw loosening but stock abutment and gold cast abutment has no effect in screw loosening but in our study stock abutment has little effect in screw loosening .

A key novel finding of this study is that while CAD/CAM technology offers individualized prosthetic solutions, it demonstrated significantly higher RTL compared to stock abutments after cyclic loading (*p* < 0.05) and this is in agreement with Jorge JR et al. [74], While some literature suggests parity between these fabrication methods, our results align with the theory that stock abutments possess superior fit and a higher volume of material at the interface. This suggests that the milling process for custom abutment interfaces may not yet match the industrial precision of prefabricated stock components, potentially leading to increased axial displacement and subsequent preload loss under dynamic stress [75, 76].

The superior torque maintenance observed in stock abutments compared to CAD/CAM counterparts may be attributed to the high-precision manufacturing tolerances of factory-milled components. Stock abutments are produced under standardized industrial conditions, which typically result in a more intimate interface fit and reduced microgaps at the implant-abutment junction. In contrast, CAD/CAM abutments, while offering customized emergence profiles, may exhibit slight seating discrepancies or surface irregularities arising from the milling process. These microscopic fit variations can influence micromotion and the settling effect during cyclic loading, ultimately leading to greater torque dissipation.

On the other hand our result disagree with study’s by Paek et al. [77] who reported that after the application of dynamic cyclic loading, there is no significant difference in screw loosening with the titanium stock abutment or titanium CAD/CAM abutment and also disagree with Klongbunjit D et al., [78] who concluded that customized titanium abutments, hybrid abutments and straight titanium abutments are not significantly different in terms of removal torque values after fatigue testing.

Our results indicate that the internal hexagonal connection provides superior screw joint stability compared to the trilobe design following mechanical loading. This study contributes new evidence suggesting that the six-point hexagonal indexing offers enhanced protection against the lateral flexural stresses encountered during the 150,000 cycles of simulated mastication [79, 80]. In contrast, the trilobe configuration’s three-point design appeared more susceptible to suboptimal load distribution, which likely accelerated screw loosening.

The observed superiority of the internal hexagon connection in torque maintenance can be mechanistically explained by its geometric configuration. The hexagonal design features six contact points that provide a larger surface area for anti-rotational resistance compared to the three-point contact system of the trilobe connection. Under cyclic loading, the hexagonal interface facilitates a more equitable distribution of lateral and off-axis stresses across the implant-abutment junction.

Conversely, the trilobe design, characterized by wider spans between indexing lobes, may experience higher localized stress concentrations (point loading) during dynamic function. These localized stresses can promote infinitesimal micromotion at the interface, accelerating the ‘settling effect’ and the subsequent dissipation of screw preload. This finding is consistent with finite element analysis (FEA) studies showing that connection geometry directly influences the magnitude of stress transferred to the abutment screw.

One limitation of the initial data processing was the reliance on independent and paired t-tests. While these tests provided insights into the changes within individual groups, they did not account for the interaction between the type of implant-abutment connection and the mechanical loading over time. To address this, a two-way repeated measures ANOVA was subsequently implemented, which reduced the risk of Type II errors and confirmed the statistical validity of the observed differences in torque loss across the tested groups.

Based on the findings of this study, the null hypothesis which stated there would be no difference in torque loss between connection types or abutment fabrication methods is rejected. Significant differences in removal torque loss (RTL) were observed, particularly favoring the internal hexagonal connection and stock abutments following mechanical loading.

The present investigation was conducted by the same research group that previously evaluated the influence of stock and CAD/CAM abutments on peri-implant strain development and screw loosening [40]. Although similar laboratory equipment and experimental procedures were employed, the current study addressed a different scientific objective and generated an independent dataset. All measurements, analyses, and findings reported herein were produced specifically for the present investigation.

## Conclusions

Within the limitations of this study, it can be concluded that :

1. Both internal trilobe and internal hexagonal implant-abutment connections experience removal torque loss following cyclic loading. However, the internal hexagonal connection demonstrated superior screw joint stability and higher resistance to loosening compared to the trilobe configuration.
2. Additionally, Within the limitations of this study, stock abutments demonstrated superior torque maintenance and screw joint stability compared to custom CAD/CAM abutments after simulated cyclic loading. Clinically, these findings suggest that utilizing internal hexagonal connections with stock abutments may optimize long-term prosthetic success by maintaining higher preload and reducing the incidence of mechanical complications in high-load dental restorations.

## Limitations and recommendations

- The observed differences emphasize that for high-load posterior restorations, the combination of a hexagonal internal connection and a stock abutment may provide the most predictable mechanical outcome.
- However, as this was an in vitro study limited to 150,000 cycles (simulating approximately one year of clinical use), further long-term clinical trials are required to evaluate the biological response of surrounding tissues to these mechanical variances.
- It should be noted that this study utilized removal torque as the primary outcome measure. While removal torque is a reliable surrogate for evaluating the maintenance of the screw joint over time, it is an indirect measure. Factors such as the friction coefficient of the screw threads and the ‘settling effect’ influence these values, and they do not represent a direct measurement of the tensile preload force.
- “A key limitation of the present study is the absence of a direct quantitative assessment of the interface fit, such as through micro-computed tomography (micro-CT) or scanning electron microscopy (SEM). While our data clearly indicate a difference in torque loss between groups, we cannot definitively correlate these findings to specific manufacturing tolerances or seating discrepancies. Future research should combine mechanical loading with high-resolution imaging to further elucidate the relationship between interface fit and screw joint stability.”
- The study may have simplified the clinical scenarios, not accounting for various factors that can influence screw loosening, such as occlusal forces, bone density, and patient habits.
- In this study, specimens were subjected to mechanical cyclic loading under room temperature conditions without thermocycling. While this approach allows for a controlled comparison of the mechanical stability of different connection types, it does not fully replicate the complex intraoral environment. In the oral cavity, constant fluctuations in temperature and humidity can induce thermal stresses and potential ‘aging’ of the implant-abutment interface, which may further influence the settling effect and screw loosening. The omission of thermocycling should be considered a limitation of this study; therefore, the results describe the mechanical behavior of these joints under isolated loading conditions. Future research incorporating both thermocycling and mechanical loading is necessary to provide a more comprehensive prediction of long-term clinical performance.

## Data Availability

The data supporting the findings of this study are available upon reasonable request from the corresponding author

## Abbreviations

CAD/CAM: Computer-Aided manufacturing /Computer Aided designing
NCm: Newton-Centimeter
RTL: Removal torque loss
GPa: Gigapascals

## References

[1] Abraham CM. A Brief Historical Perspective on Dental Implants, Their Surface Coatings and Treatments. Open Dent. 2014;16:50–5. 10.2174/1874210601408010050 PMC4040928 24894638

[2] Singer SL, Henry PJ, Liddelow G, Rosenberg I. Long-term follow-up of implant treatment for oligodontia in an actively growing individual: a clinical report. J Prosthet Dent. 2012;108:279–85. 23107235 10.1016/S0022-3913(12)60176-0

[3] JULIA-GABRIELA WITTNEBEN TJ, HANS-PETER, WEBER & UR S BRAGGER. Screw retained vs. cement retained implant-supported fixed dental prosthesis. Periodontol 2000. 2017;73:141–51. 28000276 10.1111/prd.12168

[4] Sammour SR, El-Sheikh MM, El-Gendy AA. Effect of implant abutment connection designs, and implant diameters on screw loosening before and after cyclic loading: in-vitro study. Dent materials: official publication Acad Dent Mater. 2019;35:265–71. 10.1016/j.dental.2019.07.026 31427043

[5] Jeng M-D, Liu P-Y, Kuo J-H, Lin C-L. Load fatigue performance evaluation on two internal tapered abutment–implant connection implants under different screw tightening torques. J Oral Implantol. 2017;43:107–13. 28045598 10.1563/aaid-joi-D-16-00129

[6] Kofron MD, Carstens M, Fu C, Wen HB. In vitro assessment of connection strength and stability of internal implant-abutment connections. Clin Biomech. 2019;65:92–9. 10.1016/j.clinbiomech.2019.03.007 31005695

[7] Vetromilla BM, Brondani LP, Pereira-Cenci T, Bergoli CD. Influence of different implant-abutment connection designs on the mechanical and biological behavior of single-tooth implants in the maxillary esthetic zone: A systematic review. J Prosthet Dent. 2019;121(3):398–403. e393. 30477924 10.1016/j.prosdent.2018.05.007

[8] Menacho-Mendoza E, Cedamanos-Cuenca R, Díaz-Suyo A. Stress analysis and factor of safety in three dental implant systems by finite element analysis. Saudi Dent J. 2022;34(7):579–84. 36267532 10.1016/j.sdentj.2022.08.006 PMC9577351

[9] Larrucea Verdugo C, Jaramillo Núñez G, Acevedo Avila A, Larrucea San Martín C. Microleakage of the prosthetic abutment/implant interface with internal and external connection: In vitro study. Clin Oral Implants Res. 2014;25:1078–83. 23822097 10.1111/clr.12217

[10] Valvi NN, Khalikar S, Mahale K, Rajguru V, Mahajan S, Tandale U. Evolving interfaces: A comprehensive review of implant-abutment connections. Int Dent J Student Res. 2024;12:1–14.

[11] Shah KK, Sivaswamy V. A literature review on implant abutment types, materials, and fabrication processes. J Long Term Eff Med Implants. 2023;33:57–66. 10.1615/JLongTermEffMedImplants.2022042720 36382705

[12] Muley N, Prithviraj D, Gupta V. Evolution of external and internal implant to abutment connection. Int J Oral Implantol Clin Res. 2012;3:122–9.

[13] Vinhas AS, Aroso C, Salazar F, López-Jarana P, Ríos-Santos JV, Herrero-Climent M. Review of the mechanical behavior of different implant–abutment connections. Int J Environ Res Public Health. 2020;17:8685–95. 33238476 10.3390/ijerph17228685 PMC7700386

[14] Pera F, Menini M, Bagnasco F, Mussano F, Ambrogio G, Pesce P. Evaluation of internal and external hexagon connections in immediately loaded full-arch rehabilitations: A within‐person randomized split‐mouth controlled trial with a 3‐year follow‐up. Clin Implant Dent Relat Res. 2021;23:562–7. 34219356 10.1111/cid.13029 PMC8457096

[15] Salinas TJ. A change of philosophy in abutment connections. Pract Proced Aesthet Dent. 2002;14:471–471. 12242857

[16] Alonso-Pérez R, Bartolomé JF, Ferreiroa A, Salido MP, Pradíes G. Evaluation of the Mechanical Behavior and Marginal Accuracy of Stock and Laser-Sintered Implant Abutments. Int J Prosthodont. 2017;30:136–9. 10.11607/ijp.5089 28267820

[17] Elsayed M-E, Shakal E, Mostafa T. Effect of internal hex height and collar height on marginal fit of implant abutment connection after dynamic cyclic loading:(In Vitro Study). J Int Dent Medic Res. 2021;14:79–86.

[18] Kapos T, Evans C. CAD/CAM technology for implant abutments, crowns, and superstructures. Int J Oral Maxillofacial Implants. 2014;29:117–28. 10.11607/jomi.2014suppl.g2.3 24660194

[19] Tramontana D. Custom Abutments for Dental Implants, Their Evolution and Uses: A Narrative Review. PQDT-Global. 2022;11:1–6.

[20] de Melo Moreno AL, Dos Santos DM, de Magalhães Bertoz AP, Goiato MC. Abutment on titanium-base hybrid implant: A literature review. Eur J dentistry. 2023;17(02):261–9. 10.1055/s-0042-1750801 PMC10329526 36220125

[21] Khorshid HE. A Comparative Study of the Biological and Prosthetic Complications of CAD/CAM Zirconia and Conventional Cast Implant Supported Hybrid Prostheses. Adv Dent J. 2023;5(3):690–9.

[22] Shenava A. Failure mode of implant abutment connections: An overview. J Dent Med Sci. 2013;11:32–5.

[23] Keith L, Guzaitis KLK, Marlos AG, Viana. Effect of repeated screw joint closing and opening cycles on implant prosthetic screw reverse torque and implant and screw thread morphology. J Prosthet Dent. 2011;106:159–159. 21889002 10.1016/S0022-3913(11)60115-7

[24] Martin WC, Woody RD, Miller BH, Miller AW. Implant abutment screw rotations and preloads for four different screw materials and surfaces. J Prosthet Dent. 2001;86:24–32. 11458261 10.1067/mpr.2001.116230

[25] Wittneben JG, Joda T, Weber HP, Brägger U. Screw retained vs. cement retained implant-supported fixed dental prosthesis. Periodontol 2000. 2017;73:141–51. 28000276 10.1111/prd.12168

[26] Siamos G, Winkler S, Boberick KG. The relationship between implant preload and screw loosening on implant-supported prostheses. J Oral Implantol. 2002;28:67–73. 12498448 10.1563/1548-1336(2002)028<0067:TRBIPA>2.3.CO;2

[27] Sailer I, Philipp A, Zembic A, Pjetursson BE, Hämmerle CH, Zwahlen M. A systematic review of the performance of ceramic and metal implant abutments supporting fixed implant reconstructions. Clin Oral Implants Res. 2009;20:4–31. 19663946 10.1111/j.1600-0501.2009.01787.x

[28] Euán R, Figueras-Álvarez O, Cabratosa-Termes J, Oliver-Parra R. Marginal adaptation of zirconium dioxide copings: influence of the CAD/CAM system and the finish line design. J Prosthet Dent. 2014;112:155–62. 24445027 10.1016/j.prosdent.2013.10.012

[29] Yeung S, Aghvinian R, Aalam A-A, Jivraj S. CAD-CAM approach to designing and fabricating a low-maintenance metal-acrylic resin implant-supported fixed complete dental prosthesis: A clinical report. J Prosthet Dent. 2021;125:719–22. 32576372 10.1016/j.prosdent.2020.04.006

[30] Santiago Júnior O, Ferreira MVL, Huebner R. Do chewing simulators influence the test results of dental materials? Systematic review. Jaw Funct Orthop Craniofac Growth. 2023;3(1):1–18.

[31] Ranjan M, Chatterjee U, Keshri M, Kumar M, Rani P. Occlusal in implant Prosthesis: Book Rivers. 2021.

[32] Rachel A, Sheridan AMD, Alexandra B, Plonka H-L, Wang. The Role of Occlusion in Implant Therapy: A Comprehensive Updated Review. Implant Dent. 2016;25:829–38. 27749518 10.1097/ID.0000000000000488

[33] Khraisat A, Hashimoto A, Nomura S, Miyakawa O. Effect of lateral cyclic loading on abutment screw loosening of an external hexagon implant system. J Prosthet Dent. 2004;91:326–34. 15116033 10.1016/j.prosdent.2004.01.001

[34] Ilić G, Vulović S, Bukorović J, Dragović M, Marković A, Todorović A. The impact of abutment type on abutment screw removal torque value after experimental aging. J prosthodontics: official J Am Coll Prosthodontists. 2024;10:13978–85. 10.1111/jopr.13978 39476183

[35] Zarbakhsh A, Alaei M, Bitaraf T. Evaluation of the effect of zirconia and titanium abutments on microleakage of implant-abutment interface under oblique cyclic loading in vitro. J Res Dent Maxillofac Sci. 2020;5:15–20.

[36] Cerutti-Kopplin D, Neto DJR, do Valle AL, Pereira JR, Pinho LGND. Reverse torque values in indexed abutments. J Res Dent. 2013;1:192–183.

[37] Buser D, Sennerby L, De Bruyn H. Modern implant dentistry based on osseointegration: 50 years of progress, current trends and open questions. Periodontol 2000. 2017;73:7–21. 28000280 10.1111/prd.12185

[38] Vetromilla BM, Brondani LP, Pereira-Cenci T, Bergoli CD. Influence of different implant-abutment connection designs on the mechanical and biological behavior of single-tooth implants in the maxillary esthetic zone: A systematic review. J Prosthet Dent. 2019;121:398–403. 30477924 10.1016/j.prosdent.2018.05.007

[39] Alhroob KH, Alsabbagh MM, Alsabbagh AY. Effect of the use of a surgical guide on heat generation during implant placement: A comparative in vitro study. Dent Med probl. 2021;58:55–9. 33754500 10.17219/dmp/127605

[40] Mostafa MH, El Gendy AA, Elfeky AF. Influence of conical hybrid stock and computer-aided design/computer-aided manufacturing abutments on the strain developed around implant and the screw loosening. Tanta Dent J. 2024;21(3):265–74.

[41] Lerner H. Experimental and Clinical Results to Support Digital Workflows in Implant Dentistry [disseration]. ProQuest Disseration & Theses Global. 2021.

[42] Chee WW, Duncan J, Afshar M, Moshaverinia A. Evaluation of the amount of excess cement around the margins of cement-retained dental implant restorations: the effect of the cement application method. J Prosthet Dent. 2013;109(4):216–21. 23566601 10.1016/S0022-3913(13)60047-5

[43] Gultekin P, Gultekin BA, Aydin M, Yalcin S. Cement selection for implant-supported crowns fabricated with different luting space settings. J Prosthodontics: Implant Esthetic Reconstr Dentistry. 2013;22(2):112–9. 10.1111/j.1532-849X.2012.00912.x 23387964

[44] Nengpichong Haokip ST, Pronob Sanyal. Shubha Kamnoor To determine the effect of plasma nitriding treatment 56 on screw loosening and surface topography of different 78 implant-abutment screw systems with and without thermocycling: An in vitro study. J Indian Prosthodont Soc. 2023;23:285–93. 37929368 10.4103/jips.jips_147_23 PMC10467315

[45] Attiah EMN, AlGendy AA, Mostafa TMN. Effect of dynamic cyclic loading on screw loosening of retightened versus new abutment screw in both narrow and standard implants (in-vitro study). Int J Implant Dent. 2020;6:1–12. 32720011 10.1186/s40729-020-00229-3 PMC7385049

[46] Çötert İ, Ulusoy M, Türk A. In–Vitro Comparison of Screw Loosening, Fracture Strength and Failure Mode of Implant–Supported Hybrid–Abutment Crowns and Screwmentable Crowns Manufactured with Different Materials. Niger J Clin Pract. 2025;28(4):461–70. 40289002 10.4103/njcp.njcp_772_23

[47] Nawafleh N, Hatamleh M, Elshiyab S, Mack F. Lithium disilicate restorations fatigue testing parameters: a systematic review. J prosthodontics: official J Am Coll Prosthodontists. 2016;25:116–26. 10.1111/jopr.12376 26505638

[48] Scarano A, Quaranta M, Traini T, Piattelli M, Piattelli A. SEM and fractography analysis of screw thread loosening in dental implants. Int J ImmunoPathol Pharmacol. 2007;20(1suppl):19–22. 17897496 10.1177/039463200702001s05

[49] Kim E-S, Shin S-Y. Influence of the implant abutment types and the dynamic loading on initial screw loosening. J Adv Prosthodont. 2013;5(1):21. 23509006 10.4047/jap.2013.5.1.21 PMC3597922

[50] Lee CK, Karl M, Kelly JR. Evaluation of test protocol variables for dental implant fatigue research. Dent materials: official publication Acad Dent Mater. 2009;25:1419–25. 10.1016/j.dental.2009.07.003 19646746

[51] Pellizzer EP, Verri FR, Falcón-Antenucci RM, Goiato MC, Gennari Filho H. Evaluation of different retention systems on a distal extension removable partial denture associated with an osseointegrated implant. J Craniofac Surg. 2010;21:727–34. 20485037 10.1097/SCS.0b013e3181d8098a

[52] Trivedi S. Finite element analysis: A boon to dentistry. J Oral Biol Craniofac Res. 2014;4:200–3. 25737944 10.1016/j.jobcr.2014.11.008 PMC4306993

[53] Abdel-Halim M, Issa D, Chrcanovic BR. The impact of dental implant length on failure rates: A systematic review and meta-analysis. Materials. 2021;14:3972–83. 34300891 10.3390/ma14143972 PMC8307721

[54] Gracis S, Michalakis K, Vigolo P, von Vult P, Zwahlen M, Sailer I. Internal vs. external connections for abutments/reconstructions: a systematic review. Clin Oral Implants Res. 2012;23:202–16. 23062143 10.1111/j.1600-0501.2012.02556.x

[55] de França DG, Morais MH, das Neves FD, Barbosa GA. Influence of CAD/CAM on the fit accuracy of implant-supported zirconia and cobalt-chromium fixed dental prostheses. J Prosthet Dent 2015. 2015;113(1):22–8. 10.1016/j.prosdent.2014.07.010 25277028

[56] Giménez B, Özcan M, Martínez-Rus F, Pradíes G. Accuracy of a digital impression system based on parallel confocal laser technology for implants with consideration of operator experience and implant angulation and depth. Int J Oral Maxillofac Implants. 2014;29(4):853–62. 25032765 10.11607/jomi.3343

[57] Marques S, Ribeiro P, Falcão C. Digital Impressions in Implant Dentistry: A Literature Review. Int J Environ Res Public Health. 2021;18:1020–31. 33498902 10.3390/ijerph18031020 PMC7908474

[58] Almeida e Silva JS, Erdelt K, Edelhoff D, Araújo É, Stimmelmayr M, Vieira LC. Marginal and internal fit of four-unit zirconia fixed dental prostheses based on digital and conventional impression techniques. Clin Oral Invest. 2014;18:515–23. 10.1007/s00784-013-0987-2 23716064

[59] Tajti P, Solyom E, Czumbel LM, Szabó B, Fazekas R, Németh O. Monolithic zirconia as a valid alternative to metal-ceramic for implant-supported single crowns in the posterior region: A systematic review and meta-analysis of randomized controlled trials. J Prosthet Dent. 2023;132:881–9. 37349158 10.1016/j.prosdent.2023.05.006

[60] Maria Menini FB, Calimodio I, Nicolò, Di Tullio F, Delucchi D, Baldi. Et al: Influence of Implant Thread Morphology on Primary Stability: A Prospective Clinical Study. *Biomed Res Int.* 2020;5:697–703. 10.1155/2020/6974050 PMC7426766 32802868

[61] Jividen G Jr, Misch CE. Reverse torque testing and early loading failures: help or hindrance? J Oral Implantol. 2000;26:82–90. 11831335 10.1563/1548-1336(2000)026<0082:RTTAEL>2.3.CO;2

[62] Pardal-Peláez B, Montero J. Preload loss of abutment screws after dynamic fatigue in single implant-supported restorations. A systematic review. J Clin Exp Dent. 2017;9:1355–61. 10.4317/jced.54374 PMC5741850 29302289

[63] El-Sheikh MAY, Mostafa TMN, El-Sheikh MM. Effect of different angulations and collar lengths of conical hybrid implant abutment on screw loosening after dynamic cyclic loading. Int J implant dentistry. 2018;4(1):39. 10.1186/s40729-018-0149-z PMC6275154 30506525

[64] Mancini G, Cura F, Ormanier Z, Carinci F. Efficacy of a new implant-abutment connection to minimize microbial contamination: An in vitro study. Oral Implantol. 2016;9:99–105. 10.11138/orl/2016.9.3.099 PMC5159928 28042437

[65] Rosentritt M, Behr M, van der Zel JM, Feilzer AJ. Approach for valuating the influence of laboratory simulation. Dent materials: official publication Acad Dent Mater. 2009;25:348–52. 10.1016/j.dental.2008.08.009 18829097

[66] Kanazawa M, Minakuchi S, Hayakawa I, Hirano S, Uchida T. In vitro study of reduction of stress transferred onto tissues around implants using a resilient material in maxillary implant overdentures. J Med Dent Sci. 2007;54(1):17–23. 19845131

[67] Byrne D, Jacobs S, O’Connell B, Houston F, Claffey N. Preloads generated with repeated tightening in three types of screws used in dental implant assemblies. J Prosthodont. 2006;15:164–71. 16681498 10.1111/j.1532-849X.2006.00096.x

[68] Yi-Han Su B-YP, Wang P-D. Sheng-Wei Feng Evaluation of the implant stability and the marginal bone level changes during the first three months of dental implant healing process: A prospective clinical study. J Mech Behav Biomed Mater. 2020;110:103899–910. 32957204 10.1016/j.jmbbm.2020.103899

[69] Shin H-M, Huh J-B, Yun M-J, Jeon Y-C, Chang BM, Jeong C-M. Influence of the implant-abutment connection design and diameter on the screw joint stability. J Adv Prosthodont. 2014;6:126–32. 24843398 10.4047/jap.2014.6.2.126 PMC4024557

[70] Bakaeen LG, Winkler S, Neff PA. The effect of implant diameter, restoration design, and occlusal table variations on screw loosening of posterior single-tooth implant restorations. J Oral Implantology. 2001;27(2):63–72. 12498429 10.1563/1548-1336(2001)027<0063:TEOIDR>2.3.CO;2

[71] Bickford JH. An introduction to the designand behavior of bolted joints. Volume 3. New York: Marcel Dekker Inc; 1995. pp. 515–64.

[72] Yi Y, Heo SJ. Comparison of CAD/CAM abutment and prefabricated abutment in Morse taper internal type implant after cyclic loading: Axial displacement, removal torque, and tensile removal force. J Adv Prosthodont. 2019;11(6):305–12. 31897269 10.4047/jap.2019.11.6.305 PMC6933047

[73] Kim E-S, Shin S-Y. Influence of the implant abutment types and the dynamic loading on initial screw loosening. J Adv Prosthodont. 2013;5(1):21–8. 23509006 10.4047/jap.2013.5.1.21 PMC3597922

[74] Jorge JR, Barao VA, Delben JA, Assuncao WG. The role of implant/abutment system on torque maintenance of retention screws and vertical misfit of implant-supported crowns before and after mechanical cycling. Int J Oral Maxillofac Implants. 2013;28(2):415–22. 23527343 10.11607/jomi.2727

[75] Alonso-Pérez R, Bartolomé JF, Ferreiroa A, Salido MP, Pradíes G. Evaluation of the Mechanical Behavior and Marginal Accuracy of Stock and Laser-Sintered Implant Abutments. Int J Prosthodont 2017;30(2):136–8. 28267820 10.11607/ijp.5089

[76] Lops D, Meneghello R, Sbricoli L, Savio G, Bressan E, Stellini E. Precision of the Connection Between Implant and Standard or Computer-Aided Design/Computer-Aided Manufacturing Abutments: A Novel Evaluation Method. Int J Oral Maxillofac Implants. 2018;33(1):23–30. 29340342 10.11607/jomi.5411

[77] Paek J, Woo YH, Kim HS, Pae A, Noh K, Lee H, Kwon KR. Comparative Analysis of Screw Loosening With Prefabricated Abutments and Customized CAD/CAM Abutments. Implant Dent. 2016;25(6):770–4. 27571352 10.1097/ID.0000000000000481

[78] Klongbunjit D, Aunmeungtong W. Implant-abutment screw removal torque values between customized titanium abutment, straight titanium abutment, and hybrid zirconia abutment after a million cyclic loading: an in vitro comparative study. nt J Implant Dent 2021;7(1):80–98. 10.1186/s40729-021-00378-z PMC8487932 34604929

[79] Choi S, Kang YS, Yeo I-SL. Influence of implant–abutment connection biomechanics on biological response: a literature review on interfaces between implants and abutments of titanium and zirconia. Prosthesis. 2023;5(2):527–38.

[80] Yuya Sasada DLC. Implant-Abutment Connections: A Review of Biologic Consequences and Peri-implantitis Implications. Int J Oral Maxillofac Implants. 2017;32:1296–307. 29140374 10.11607/jomi.5732

